# Behavioral comorbidities treatment by fecal microbiota transplantation in canine epilepsy: a pilot study of a novel therapeutic approach

**DOI:** 10.3389/fvets.2024.1385469

**Published:** 2024-06-21

**Authors:** Antja Watanangura, Sebastian Meller, Nareed Farhat, Jan S. Suchodolski, Rachel Pilla, Mohammad R. Khattab, Bruna C. Lopes, Andrea Bathen-Nöthen, Andrea Fischer, Kathrin Busch-Hahn, Cornelia Flieshardt, Martina Gramer, Franziska Richter, Anna Zamansky, Holger A. Volk

**Affiliations:** ^1^Department of Small Animal Medicine and Surgery, University of Veterinary Medicine Hannover, Hannover, Germany; ^2^Center for Systems Neuroscience (ZSN), Hannover, Germany; ^3^Veterinary Research and Academic Service, Faculty of Veterinary Medicine, Kasetsart University, Nakhon Pathom, Thailand; ^4^Tech4Animals Lab, Information Systems Department, University of Haifa, Haifa, Israel; ^5^Gastrointestinal Laboratory, Department of Small Animal Clinical Sciences, College of Veterinary Medicine and Biomedical Sciences, Texas A&M University, TX, United States; ^6^Department of Molecular Microbiology and Immunology, School of Medicine, University of Missouri, Columbia, MO, United States; ^7^Tierarztpraxis Dr A. Bathen-Nöthen, Cologne, Germany; ^8^Centre for Clinical Veterinary Medicine, Ludwig-Maximilians-Universität München, Munich, Germany; ^9^Small Animal Clinic, Tierklinik Posthausen, Germany; ^10^Department of Pharmacology, Toxicology, and Pharmacy, University of Veterinary Medicine Hannover, Hannover, Germany

**Keywords:** fecal microbiota transplantation, canine idiopathic epilepsy, behavioral comorbidities, gastrointestinal microbiota, microbiota-gut-brain axis

## Abstract

**Introduction:**

Anxiety and cognitive dysfunction are frequent, difficult to treat and burdensome comorbidities in human and canine epilepsy. Fecal microbiota transplantation (FMT) has been shown to modulate behavior in rodent models by altering the gastrointestinal microbiota (GIM). This study aims to investigate the beneficial effects of FMT on behavioral comorbidities in a canine translational model of epilepsy.

**Methods:**

Nine dogs with drug-resistant epilepsy (DRE) and behavioral comorbidities were recruited. The fecal donor had epilepsy with unremarkable behavior, which exhibited a complete response to phenobarbital, resulting in it being seizure-free long term. FMTs were performed three times, two weeks apart, and the dogs had follow-up visits at three and six months after FMTs. Comprehensive behavioral analysis, including formerly validated questionnaires and behavioral tests for attention deficit hyperactivity disorder (ADHD)- and fear- and anxiety-like behavior, as well as cognitive dysfunction, were conducted, followed by objective computational analysis. Blood samples were taken for the analysis of antiseizure drug (ASD) concentrations, hematology, and biochemistry. Urine neurotransmitter concentrations were measured. Fecal samples were subjected to analysis using shallow DNA shotgun sequencing, real-time polymerase chain reaction (qPCR)-based Dysbiosis Index (DI) assessment, and short-chain fatty acid (SCFA) quantification.

**Results:**

Following FMT, the patients showed improvement in ADHD-like behavior, fear- and anxiety-like behavior, and quality of life. The excitatory neurotransmitters aspartate and glutamate were decreased, while the inhibitory neurotransmitter gamma-aminobutyric acid (GABA) and GABA/glutamate ratio were increased compared to baseline. Only minor taxonomic changes were observed, with a decrease in Firmicutes and a *Blautia_A* species, while a *Ruminococcus* species increased. Functional gene analysis, SCFA concentration, blood parameters, and ASD concentrations remained unchanged.

**Discussion:**

Behavioral comorbidities in canine IE could be alleviated by FMT. This study highlights FMT’s potential as a novel approach to improving behavioral comorbidities and enhancing the quality of life in canine patients with epilepsy.

## 1 Introduction

Epilepsy is a common neurological disease in humans and dogs (1). It is commonly known that attention deficit hyperactivity disorder (ADHD)-, fear, and anxiety behavior, as well as cognitive dysfunction are epilepsy comorbidities in both species with an assumed bidirectional relationship (2). Neurobehavioral comorbidities impact the quality of life for owners and their canine companions, mirroring the challenges faced in human epilepsy (3–5). Management of the comorbidities in human and canine epilepsy, via nutrition, medication and through behavioral modifications, is still in its infancy (2). Therefore, the search for well-tolerated and effective therapeutic strategies to mitigate behavioral comorbidities and cognitive dysfunctions are needed.

Epilepsy is usually treated with antiseizure drugs (ASDs) (1), but not all cases respond adequately to ASDs. In dogs, drug-resistant epilepsy (DRE) is defined by the International Veterinary Epilepsy Task Force, similarly to the International League Against Epilepsy, as a failure of adequate trials of two well-tolerated, appropriately chosen ASDs to achieve sustained seizure freedom (6). Based on this definition, two-thirds of dogs with epilepsy are classified as drug-resistant (7). In this population, the behavioral comorbidities are present to a greater extent (8). To date, however, it has still not been clearly elucidated, in its complete granularity, how these behavioral comorbidities and cognitive dysfunctions develop and progress in the affected patients during epileptogenesis or disease progression and its treatment. Vice versa, it is not clear how epilepsy develops in patients with these comorbidities (9).

A promising link between behavioral comorbidities and epilepsy could be the microbiota-gut-brain axis (MGBA) (10). The MGBA refers to a bidirectional communication between the gastrointestinal microbiota (GIM) and the brain. The term MGBA summarizes many pathways including the enteric nervous system as part of the autonomic nervous system, hypothalamic pituitary adrenal axis, inflammatory pathway, immune and neuroendocrine system, as well as neural tracts (10). Fecal microbiota transplantation (FMT) is considered an effective procedure to recalibrate GIM by administering fecal material from an unaffected donor to a diseased patient (11). In the last decade, several neurological and neuropsychiatric disorders, including Alzheimer’s disease, Parkinson’s disease, autism spectrum disorders and epilepsy have been reported to have GIM alterations (10).

These findings raise the question as to whether FMT can normalize the GIM and consequently improve clinical signs in these patients. Based on the principle of the MGBA, multiple studies have shown that behavior can be transplanted from affected to non-affected animals and even across species barriers. A preclinical study showed that depression, anhedonia and anxiety-like behavior from human patients could be transferred to rats by FMT (12). Another study demonstrated that recipient mice receiving fecal material from mice with chronic stress, exhibited similar anxiety- and depression-like behavior as the donors (13).

In the current pilot study, the aims were to determine the effects of FMT in dogs with DRE as a potential novel treatment on behavioral comorbidities and cognitive dysfunction.

## 2 Materials and methods

### 2.1 Study design

**Figure 1:**
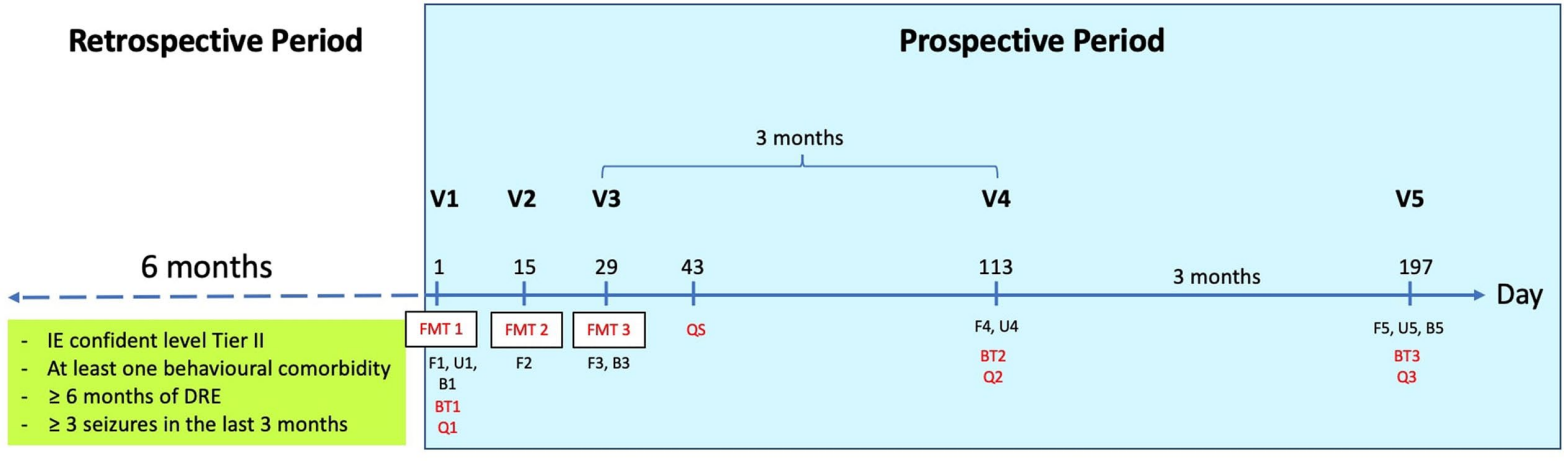
The diagram demonstrates the study plan. Baseline seizure frequency was evaluated retrospectively over a six-month period. Behavior was assessed prior to the first fecal microbiota transplant (FMT). Seizure and behavior were then monitored on a three-monthly basis. At V1, all sample collections and tests were performed before the first FMT. DRE, drug-resistant epilepsy; V, visit; FMT, fecal microbiota transplantation; F, fecal sample; U, urine sample; B, blood sample; BT, behavioral test; Q, questionnaire; QS, questionnaire for FMT effects and side effects.

The open-label six-month prospective pilot study included a total of five on-site visits (V). FMT was performed three times 2 weeks apart (V1-V3), and follow-up appointments were scheduled at 3 months (V4) and 6 months (V5) after FMT (Figure 1). The study was approved by the Lower Saxony State Office for Consumer Protection and Food Safety, Germany (LAVES; ID 33.8–42,502-05-20A539).

### 2.2 Dogs

Dogs with drug-resistant idiopathic epilepsy (IE) Tier II confidence level (14) and at least one behavioral comorbidity, such as ADHD or fear- and anxiety-like behavior (including separation anxiety, fear aggression, fear of loud noise, other dogs, strangers, and sudden movements), were recruited in the prospective phase of the study. DRE is defined as the inability to achieve sustained seizure freedom despite undergoing sufficient trials of two tolerated, appropriately selected, and properly administered antiepileptic drug regimens (6). Criteria are shown in Table 1. Owners were required not to change their dogs’ diets and treats throughout the study and to provide written informed consent for participation.

**Table 1:** Inclusion and exclusion criteria of participants and donor candidates.

Participant’s criteria	
Inclusion criteria	Exclusion criteria
- Having at least one behavioral comorbidity - At least 6 months of DRE - At least three generalized seizures in the last 3 months under unchanged ASDs and dosages - One of the ASDs had to be PB - Serum ASDs’ concentration in therapeutic ranges - No changes of diets and treats in the last 3 months - In case the dogs received any supplements, the supplement has to be given at least 3 months regularly without changing of dosage.	- Being treated with antibiotics in the last 6 months - Being treated with any drug which could potentially interact with ASDs - Chronic GI diseases - Any allergy including food allergy - Cardiac, renal or hepatic failures - Any surgery in the last 3 months - Females known or suspected to be pregnant or lactating
Donor candidate’s criteria	
Inclusion criteria	Exclusion criteria
- No previous or current behavioral changes - IE Tier II confidence level - Completely healthy apart from IE - Being treated only with PB - Seizure-freedom for more than a year - No changes of diets and treats in the last 3 months	- Showing behavioral changes or aggression - Being treated with antibiotics or any other medication that could interfere with GIM in the last 3 months - Any surgery in the last 3 months - Chronic GI diseases - Any allergy including food allergy - Cardiac, renal or hepatic failures - Females known or suspected to be pregnant or lactating - Abnormal Dysbiosis Index (>0)

DRE, drug-resistant epilepsy; ASDs, antiseizure drugs; PB, phenobarbital; GI, gastrointestinal; IE, idiopathic epilepsy.

### 2.3 Fecal material

FMT donor candidates with well-controlled IE of Tier II confidence level (14) and unremarkable behavior were screened based on the criteria presented in Table 1. Their fecal samples were sent to the Gastrointestinal Laboratory at Texas A&M University, TX, United States, for Dysbiosis Index (DI) assessment and enteropathogen examination. Endoparasite screening was performed by the Institute of Parasitology of the University of Veterinary Medicine Hannover, Hannover, Germany. These tests were carried out to evaluate the GIM and rule out any possibility of infectious pathogen transfer. Only one dog that passed all tests was selected as the fecal donor to minimize confounding factors.

### 2.4 Fecal material preparation

Fresh fecal material from the donor was collected and prepared for long-term storage. For preparation, 25 g of fecal material was mixed with 22 mL normal saline and 3 mL glycerol in 50 mL plastic tubes (Sarstedt^®^, Nümbrecht, Germany) and stored at −20^°^C for maximum 2 months. On the day of the FMT, the materials were thawed at room temperature for 2–3 h and blended to achieve a smooth consistency. The blended materials were then drawn using 50 mL syringes (Dispomed^®^, Dispomed WITT oHG, Gelnhausen, Germany).

### 2.5 Fecal microbiota transplantation

The fecal dosage was 2.5–5 g of feces per kg body weight (15) for each of the three FMT sessions, 2 weeks apart. The dogs were not fasted or induced defecation before the transplantation. However, any feces that remained within reach of the finger in the rectum were removed. For each FMT, a rectal catheter (B. Braun Melsungen AG, Melsungen, Germany), 40 cm CH 25, was lubricated with paraffin oil and connected to a prepared syringe containing the blended fecal material. The fecal material was pre-injected into the catheter to deaerate and avoid air transplantation. At least two-thirds of the catheter length was inserted through the rectum to the colon depending on the body size and how well the procedure was tolerated. The prepared syringes were attached to the catheter and the blend materials were injected into the colon. During the FMT procedure, neither sedation nor anesthesia were required. The dogs were allowed to go home directly after FMT. Feeding and activity restriction for 4–6 h after FMT were recommended to reduce the risk of defecation (15).

### 2.6 Questionnaires

The online questionnaires were sent to owners at V1, V4, and V5 (see Figure 1). These questionnaire packages contained the formerly validated canine behavioral assessment and research questionnaire (cBARQ) (16), the attention deficit hyperactivity disorder (ADHD) questionnaire (17), the canine cognitive dysfunction rating scale (CCDR) (18), and the epileptic quality of life questionnaire (EpiQoL) (4) for assessing the dogs’ potential comorbidities including fear-, anxiety-, and ADHD-like behavior, as well as cognitive dysfunction and the seizure related quality of life of the dogs including the donor and their owners. The packages also included a seizure semiology questionnaire (Supplementary file 1). Two weeks after the last FMT (V3), an online questionnaire for FMT effects and side effects (QS) was answered by the owners (Supplementary file 2).

### 2.7 Behavioral assessment

The behavioral tests including anxiety and cognition tests were conducted on the patients at V1 before the first FMT, as well as at V4 and V5 after the intervention. Dogs were not assessed in the postictal phase. The last seizure had occurred at least more than 48 h before the tests. The tests were performed in a 4×4 m testing room with some furniture and recorded by five cameras (GoPro Hero 8 Black; GoPro, Inc., San Mateo, CA, United States). The protocols used in this study are described in Supplementary file 3.

#### 2.7.1 Anxiety test

The dogs underwent three tasks, including an open field test (new environment), a separation- and stranger-directed-fear test and a second open field test (thunderstorm sound). The first open field test (new environment) and separation- and stranger-directed fear test were modified from studies by Konok and others (19) and Palestrini and others (20), while the second open field test was performed using protocols modified from a study by Gruen and others (21). The videos of the tests were analyzed using K9-Blyzer software developed by Tech4Animals,^1^ the Information Systems Department, University of Haifa, Israel. The analyzed parameters and protocols were modified from the studies by Bleuer-Elsner et al. (22) and Fux et al. (23). The method and parameters used are depicted in detail in Supplementary file 3.

#### 2.7.2 Cognition test

The cognition test consisted of two tasks including a spatial working memory task and a problem-solving task based on the modified protocol from a study by Winter and others (24). The exact times were quantified and compared.

### 2.8 Blood profiles and antiseizure drug levels

Blood samples were collected at V1 before the first FMT, at V3, and V5 for complete blood count, blood chemistry, electrolytes, bile acids, canine pancreatic lipase, serotonin and ASD (phenobarbital and potassium bromide) concentration analyses.

### 2.9 Fecal samples

The fecal samples were collected after voiding by the owners on each appointment day including V1, V2 and V3 before FMT, and V4 and V5. The samples were stored in a 5 mL plastic tubes (Sarstedt^®^, Nümbrecht, Germany) at −80°C. All samples were shipped with dry ice to the Gastrointestinal Laboratory of Texas A&M University, College Station, Texas, United States, for qPCR based DI- and short-chain fatty acid (SCFA)-analysis. The fecal DI was calculated from qPCR assays of core bacterial taxa (i.e., *Faecalibacterium*, *Turicibacter*, *Streptococcus*, *Escherichia coli*, *Blautia*, *Fusobacterium*, *Clostridium hiranonis*, and *Bifidobacterium*). The DI under 0 was considered as normobiosis (25). The concentration of SCFAs including acetate, propionate, butyrate, isobutyrate, valerate, and isovalerate were analyzed by stable isotope dilution gas chromatography–mass spectrometry assay. The fecal samples were then delivered to Diversigen, Houston, TX, United States, for metagenomics by shallow DNA shotgun sequencing including Kyoto Encyclopedia of Genes and Genomes (KEGG) modules for functional analysis. The results were used for alpha- and beta diversity analyses. The alpha diversity pertains to variations within a sample, while the beta diversity refers to differences between samples (26). Alpha diversity of each visit was evaluated by Chao1, Shannon-Wienner index and observed features. Beta diversity was evaluated by weighted and unweighted UniFrac distance measures, and principle coordinate analysis (PCoA) plots using Bray–Curtis dissimilarity. The differences in microbiota clustering between all dogs from each visit and the donor were investigated by Bray-Curtis distance metric analysis and beta-diversity with QIIME2. For functional gene analysis, KEGG orthology groups were identified by aligning them at 97% identity with the prokaryotic gene and pathway databases. The pathway database represents gene-associated essential cellular processes such as metabolism, membrane transport, signal transduction and cell cycle (27, 28). The methods of DI, SCFA evaluation and shallow DNA shotgun sequencing were previously described (29).

### 2.10 Urine samples

Midstream urine of the first urination of the day from each dog was collected by the owner in the morning of V1, V4, and V5 and then stored following established protocols (30). Urine neurotransmitters, namely, monoamine neurotransmitters (noradrenaline, adrenaline, dopamine and serotonin), amino acid neurotransmitters (glutamate, gamma-aminobutyric acid (GABA), aspartate, glycine, and serine), as well as the neurotransmitter precursors glutamine and 5-hydroxytryptophan were analyzed using high-performance liquid chromatography (HPLC) with pre-column fluorescent derivatization with the o-phthalaldehyde method, which was modified from Hörstermann’s method (31). The Fluorometer RF 20A (Shimadzu, Kyoto, Japan) was used for fluorometric detection. The methods of urine neurotransmitter analysis are described in Supplementary file 4. Creatinine concentration of each sample was also analyzed for standardization of urine concentration.

### 2.11 Seizure assessment

The owners were instructed to record their dogs’ seizure diary including seizure frequency of generalized tonic–clonic seizures, intensity, duration, clinical signs during preictal-, ictal-, postictal- and interictal phases throughout the six-month prospective period. The six-month retrospective period (6 months prior to V1) was compared to the seizure data from the prospective period at 3 months (V3-V4) and 6 months follow-up (V3-V5).

### 2.12 Statistical analysis

The data were collated and analyzed using Prism^®^ Version 9.4.1 (GraphPad Software, San Diego, CA, United States). The level of significance was set at *p*-value <0.05. The assumption of normality was tested using Shapiro–Wilk test of normality, revealing most data did not meet the assumption of normality. Hence, Wilcoxon matched-paired signed-ranks test for pairwise comparisons was used. For beta diversity (analysis of similarity, ANOSIM) multivariate statistical analysis was performed using Primer7 (Plymouth Routines in Multivariate Ecological Research Statistical Software, v7.0.13) (32). Friedman test followed by a post-hoc Dunn test was then performed on alpha diversity using JMP Pro 12 (Cary, NC, United States), and on bacterial taxa for all taxonomic levels and metagenomic data using Prism^®^ Version 9.4.1. *p*-values were adjusted for multiple comparisons with Benjamin & Hochberg FDR at a *p*-value <0.05.

## 3 Results

### 3.1 Dogs

Ten client-owned dogs with drug-resistant IE Tier II confidence level (14) were included in this study (Supplementary file 5). One dog was excluded from the study. The dog was seizure free until day 78, but then had cluster seizures, developed a neurogenic pulmonary edema and died. Data from the remaining nine dogs were utilized for behavioral analysis.

### 3.2 Donor

A seven-year-old male intact Australian Shepherd with well-controlled IE, which fulfilled all donor’s inclusion criteria (see section 2.3), was chosen as FMT donor. The dog had been treated with phenobarbital 2.2 mg/kg q12h and had been seizure-free for 1.5 years. The dog had no behavioral problems. The DI in this dog was −4.91.

### 3.3 Behavioral questionnaires

**Figure 2:**
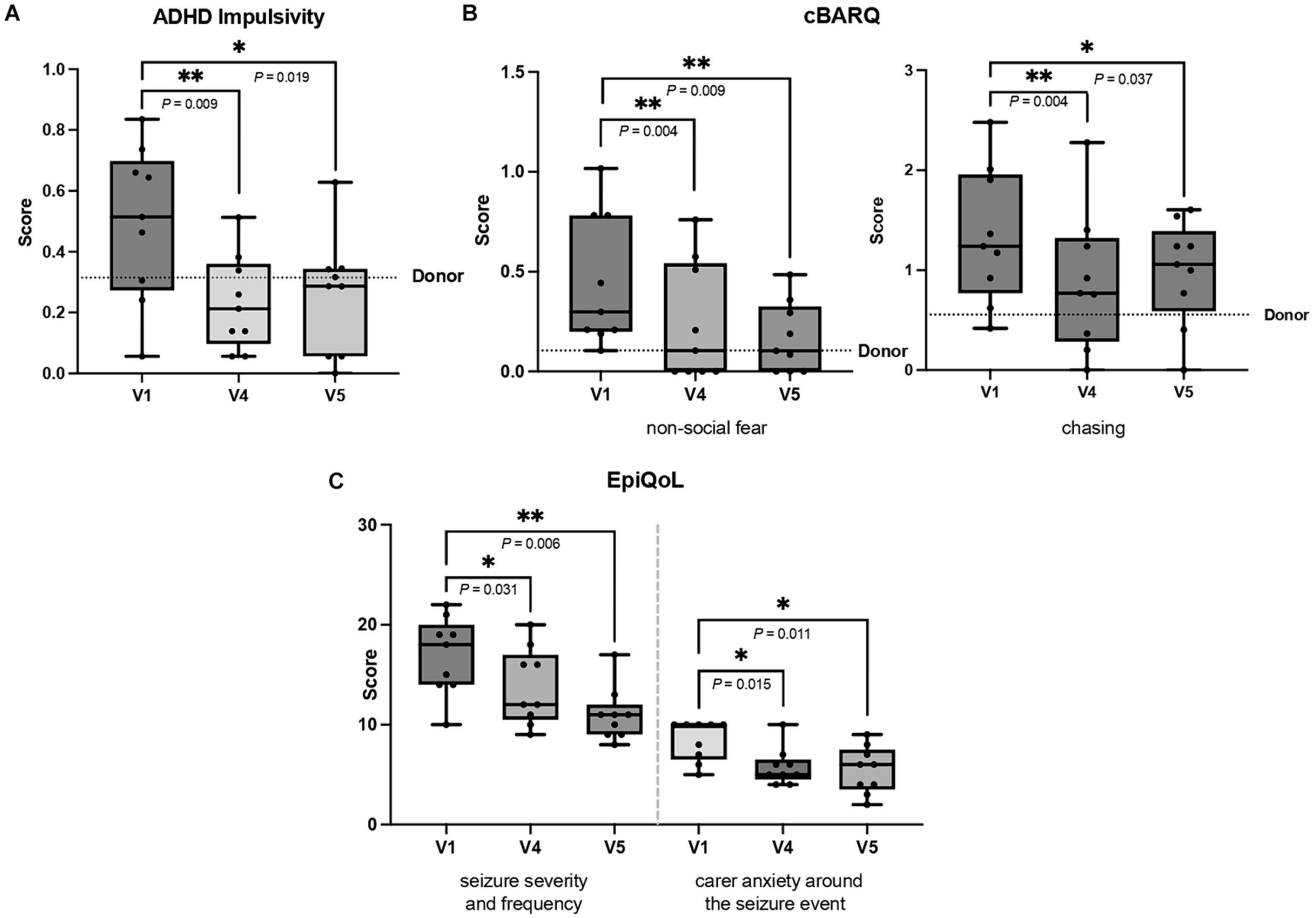
**(A–C)** The figures show significant improvement in **(A)** attention deficit hyperactivity disorder (ADHD) impulsivity and **(B)** non-social fear and chasing behavior from canine behavioral assessment and research questionnaire (C-BARQ) and **(C)** seizure severity and frequency, and carer anxiety around the seizure event from quality of life questionnaire (EpiQoL). The first questionnaire at V1 prior to FMT was compared to those at V4 (three months after FMT) and V5 (six months after FMT). Results are presented as box and whisker plots, where the horizontal line in each box represents the median, the boxes represent the interquartile range and the whiskers represent the full range of data. The dots represent individual values. The donor’s score is represented by dotted line, except for EpiQoL because the donor was seizure-free (Wilcoxon signed-rank test, *p < 0.050, **p < 0.010).

The results of questionnaires at V4 and V5 were compared to the V1 (before FMT). There was a significant improvement in the ADHD-like behavior impulsivity by V4 (*p* = 0.019) and V5 (*p* = 0.037), while no significant changes in ADHD-like behavior inattention were detected (Figure 2A). Analysis of the cBARQ showed a continuous improvement in ‘non-social fear’ by V4 (*p* = 0.004) and by V5 (*p* = 0.009). Furthermore, ‘chasing’ behavior decreased significantly at V4 (*p* = 0.004) and V5 (*p* = 0.037) (Figure 2B), another sign that the dogs were less impulsive and more under control. The EpiQoL indicated that ‘seizure severity and frequency’ improved significantly at V4 (*p* = 0.031) and V5 (*p* = 0.006). Owners also experienced reduced anxiety (‘carer anxiety around the seizure event’) at V4 (*p* = 0.015) and V5 (*p* = 0.011) (Figure 2C).

The CCDR questionnaires showed no significant changes in canine cognitive dysfunction scores between time points. However, scores at V1 (39.44 ± 4.30), V4 (36.55 ± 2.79) and V5 (37.67 ± 3.46) were below 50, indicating no signs of cognitive dysfunction (18).

### 3.4 Behavioral tests

#### 3.4.1 Anxiety test

The computational analysis was used to verify the questionnaire results and objectively evaluate the anxiety tests in the behavior lab. When comparing V4 (three months after FMT) and V5 (six months after FMT) to V1 (pre-FMT baseline), the outcomes supported the results of the questionnaire data (Table 2). The dogs were calmer and showed less impulsivity.

**Table 2:** The table demonstrates the results of three behavioral tasks, namely, open field (new environment), separation- and stranger-directed fear test (phases 1–5), and open field (thunderstorm), comparing the first visit before FMT (V1) to those 3 months after FMT (V4) and 6 months after FMT (V5).

	**Open field 1 (new environment)**					**Phase 1**					**Phase 2**				
	**Owner-dog-tester**					**Owner-dog**					**Owner-dog-stranger**				
	**V1**	**V4**	**V5**	***P*** **V1-V4**	***P*** **V1-V5**	**V1**	**V4**	**V5**	***P*** **V1-V4**	***P*** **V1-V5**	**V1**	**V4**	**V5**	***P*** **V1-V4**	***P*** **V1-V5**
TD (cm)	2560.38 ± 1405.20	2655.55 ± 1382.17	2126.79 ± 1509.96	0.191	0.248	2,168.10 ± 1144.17	1,825.44 ± 1240.36	1,440.46 ± 915.39	*0.039 ↓	*0.049 ↓	1,960.53 ± 1419.11	2,310.14 ± 1735.37	1,444.79 ± 956.29	0.273	0.150
Turn 0_30	257.44 ± 162.03	272.75 ± 166.41	194.00 ± 166.23	0.144	* 0.037↓	230.89 ± 132.54	190.25 ± 148.61	148.11 ± 109.93	*0.039 ↓	**0.082** ↓	176.66 ± 163.38	217.37 ± 179.38	120 ± 106.51	0.320	0.150
Turn 30_60	12.00 ± 7.66	13.00 ± 8.00	9.89 ± 6.35	0.500	0.262	10.11 ± 6.97	7.00 ± 2.67	6.89 ± 3.65	0.184	0.121	10.00 ± 6.40	12.50 ± 7.27	10.00 ± 7.09	0.140	0.300
Area (cm^2^)	243,356.19 ± 275,083.95	274,250.60 ± 284,304.08	229,860.11 ± 270,474.53	0.473	0.367	297,678.34 ± 265,298.05	234,135.85 ± 315,491.94	201,271.89 ± 196,068.78	0.109	0.125	191,514.98 ± 234,924.00	266,399.89 ± 300,469.65	16,138.34 ± 48,079.43	0.289	* 0.016 ↓
IU (%)	9.20 ± 6.61	10.56 ± 5.17	10.27 ± 6.76	0.500	0.455	8.60 ± 3.78	5.85 ± 5.01	5.17 ± 4.04	0.188	**0.082** ↓	6.93 ± 5.75	9.01 ± 10.60	4.43 ± 9.91	0.469	0.188
ST	0.09 ± 0.09	0.07 ± 0.03	0.05 ± 0.04	0.461	0.213	0.13 ± 0.10	0.08 ± 0.03	0.13 ± 0.10	0.250	0.285	0.08 ± 0.07	0.11 ± 0.08	0.09 ± 0.11	**0.066 ↑**	0.464
Pace (s/cm)	0.04 ± 0.04	0.04 ± 0.05	0.05 ± 0.03	0.125	0.180	0.04 ± 0.03	0.06 ± 0.05	0.08 ± 0.07	*0.012 ↑	*0.027 ↑	0.07 ± 0.12	0.08 ± 0.09	0.08 ± 0.08	0.421	0.191
Avg. speed (cm/s)	42.37 ± 23.22	44.27 ± 22.99	35.10 ± 24.74	0.231	0.248	36.14 ± 19.16	30.37 ± 20.59	24.00 ± 15.25	* 0.039 ↓	*0.049 ↓	31.76 ± 22.95	37.19 ± 28.00	23.29 ± 15.42	0.273	0.150
% OCST_O						73.27 ± 30.90	85.73 ± 20.95	86.42 ± 13.83	**0.074** ↑	0.258	79.64 ± 36.65	87.01 ± 20.83	84.98 ± 27.99	0.148	0.406
Avg. Dis_O (cm)	232.26 ± 102.05	224.26 ± 91.66	170.44 ± 83.59	***0.074 ↓***	*0.027 ↓	164.09 ± 42.25	169.48 ± 34.81	170.03 ± 63.97	0.422	0.422	190.39 ± 78.30	181.84 ± 55.74	164.12 ± 50.11	0.273	*0.082 ↓*
% OCST_S											98.55 ± 3.11	99.74 ± 0.70	99.85 ± 0.43	0.250	0.250
Avg. Dis_S (cm)											120.55 ± 50.53	106.64 ± 27.67	104.76 ± 54.95	0.273	0.285
% Duration near door 1	2.27 ± 2.93	5.33 ± 8.22	5.13 ± 8.37	0.219	0.343	5.20 ± 11.56	7.61 ± 10.53	9.18 ± 19.21	0.234	0.234	6.17 ± 6.13	6.94 ± 9.03	1.25 ± 2.15	0.500	*0.031 ↓

	**Phase 3**					**Phase 4**					**Phase 5**				
	**Dog-stranger**					**Dog alone**					**Owner-dog**				
	**V1**	**V4**	**V5**	***P*** **V1-V4**	***P*** **V1-V5**	**V1**	**V4**	**V5**	***P*** **V1-V4**	***P*** **V1-V5**	**V1**	**V4**	**V5**	***P*** **V1-V4**	***P*** **V1-V5**
TD (cm)	1662.77 ± 1515.43	1533.80 ± 893.95	1019.46 ± 592.04	0.289	0.125	2,703.68 ± 1646.90	1,989.32 ± 1173.71	1,967.31 ± 1183.33	0.320	0.218	829.12 ± 393.21	774.13 ± 339.25	687.60 ± 370.73	0.273	*0.048 ↓
Turn 0_30	94.78 ± 93.32	106.71 ± 82.73	54.22 ± 47.14	0.469	**0.060** ↓	188.44 ±171.57	183.88 ± 175.39	116 ± 102.78	0.371	**0.082** ↓	52.88 ± 31.18	51.13 ± 22.23	52.33 ± 34.34	0.273	0.424
Turn 30_60	13.00 ± 12.27	11.86 ± 9.55	7.78 ± 5.50	0.156	**0.074** ↓	18.44 ± 17.36	14.62 ± 11.13	14.00 ± 14.05	0.285	0.369	7.11 ± 2.52	8.13 ± 4.32	6.00 ± 3.54	0.320	0.113
Area (cm^2^)	58,554.90 ± 107,861.31	62,402.24 ± 54,169.09	24,863.90 ± 24,828.99	0.469	0.410	78,909.10 ± 168,182.34	148,129.51 ± 247,187.56	54,472 ± 127,282.00	0.344	**0.063** ↓	45,691.62 ± 55,926.63	35,832.32 ± 39,132.92	25,406.04 ± 21,702.13	0.231	0.213
IU (%)	8.24 ± 8.26	15.62 ± 12.93	27.73 ± 36.83	0.289	0.125	18.16 ± 39.17	4.68 ± 6.80	4.01 ± 8.51	0.344	0.188	10.73 ± 3.97	10.20 ± 2.22	11.24 ± 4.14	0.472	0.326
ST	0.11 ± 0.11	0.07 ± 0.10	0.04 ± 0.05	0.344	0.109	0.06 ± 0.05	0.02 ± 0.01	0.04 ± 0.08	0.156	0.230	0.23 ± 0.11	0.25 ± 0.09	0.36 ± 0.31	0.500	0.285
Pace (s/cm)	0.07 ± 0.06	0.05 ± 0.04	0.09 ± 0.08	0.344	0.213	0.06 ± 0.04	0.08 ± 0.10	0.08 ± 0.04	0.371	**0.082** ↑	0.02 ± 0.01	0.02 ± 0.02	0.05 ± 0.08	0.230	*0.037 ↑
Avg. speed (cm/s)	27.62 ± 25.18	25.49 ± 14.86	16.94 ± 9.84	0.289	0.150	22.48 ± 13.68	21.19 ± 11.59	16.38 ± 9.89	0.320	**0.064** ↓	56.58 ± 27.10	51.46 ± 22.00	45.16 ± 24.48	0.191	*0.037↓
% OCST_O															
Avg. Dis_O (cm)															
% OCST_S	60.51 ± 35.81	53.10 ± 37.38	37.16 ± 32.77	0.281	0.125										
Avg. Dis_S (cm)	113.11 ± 38.15	106.64 ± 38.15	106.09 ± 58.59	0.148	0.248										
% Duration near door 1	31.53 ± 32.59	37.38 ± 32.48	49.58 ± 40.92	0.344	0.213	49.34 ± 35.52	60.12 ± 37.87	57.53 ± 39.85	0.273	0.371	24.14 ± 29.80	30.23 ± 30.17	23.32 ± 32.87	0.406	0.230

	**Open field 2 (thunderstorm)**				
	**Owner-dog**				
	**V1**	**V4**	**V5**	***P*** **V1-V4**	***P*** **V1-V5**
TD (cm)	3404.37 ± 2468.93	2515.56 ± 2015.47	2501.60 ± 1882.60	0.191	0.150
Turn 0_30	338.00 ± 282.05	246 ± 251.56	250.55 ± 215.08	0.371	0.125
Turn 30_60	14.11 ± 8.81	11.50 ± 8.42	13.44 ± 14.30	0.406	0.406
Area (cm^2^)	259,210.52 ± 321,119.85	119,086.93 ± 221,201.46	227,421.62 ± 254,779.44	0.109	0.344
IU (%)	7.41 ± 7.64	5.57 ± 8.03	5.68 ± 6.09	0.281	0.234
ST	0.04 ± 0.02	0.06 ± 0.07	0.06 ± 0.07	0.472	0.468
Pace (s/cm)	0.10 ± 0.09	0.13 ± 0.11	0.14 ± 0.13	0.156	0.285
Avg. speed (cm/s)	18.88 ± 13.67	13.97 ± 11.19	13.90 ± 10.47	0.191	0.150
% OCST_O	63.02 ± 32.90	59.61 ± 34.46	66.76 ± 29.77	0.191	0.326
Avg. Dis_O (cm)	135.48 ± 34.80	121.77 ± 14.89	135.03 36.02	0.191	0.455
% OCST_S					
Avg. Dis_S (cm)					
% Duration near door 1	1.74 ± 2.97	0 ± 0.01	0.24 ± 0.46	**0.078↓**	**0.078 ↓**

The complete test methods are described in Supplementary file 3. The recorded video of each phase was analyzed using the computational “Blyzer” program. The owner- and stranger-associated values were not presented (gray boxes) if the person did not participate in the phase. In open field 1, %OCST_O was not analyzed because the owner sat on a chair near the wall and table and it was therefore not possible to draw a complete circle around the person to measure *p < 0.050 (Wilcoxon matched-paired signed-ranks test), the *p* values in bold with an asterisk (*p* < 0.050) and without an asterisk (*P* from 0.05 to 0.1) are improving values. The values in bold italics with an asterisk represent worsening values and the values in bold italics represent questionable correlation. ↓ = decrease, ↑ = increase.

TD, total distance; Turn0_30, number of turns between 0 and 30 degrees; Turn30_60, number of turns between 30 and 60 degrees; area, polygon area of the dog’s Convex hull movement; IU, intensity of use or the ratio between total movement and the square root of the area of movement; ST, straightness-net displacement distance divided by the total length of dog’s movement, Pace, the ratio of time and total distance the dog moved; Avg. speed, average speed; % OCST_O, the time spent in percentage outside the owner’s circle (1 m radius from the owner); Avg. Dis_O, average distance from owner; % OCST_S, the time spent in percentage outside the stranger’s circle (1 m radius from the stranger); % Duration near door 1, the time the dog spent within a 1-meter radius circle around door 1, expressed in percentage.

#### 3.4.2 Cognition test

The cognition tests also confirmed the questionnaire results, there being no difference in spatial working memory or problem-solving task.

### 3.5 Blood profiles and serum ASDs concentrations

No significant changes occurred in blood parameters, including serotonin and serum ASD concentrations, across all sampling time points (Supplementary file 5).

### 3.6 Fecal samples analyses

The results were presented to the genome taxonomy database (GTDB) taxonomy system. The sequence data associated with this project are deposited in the National Center for Biotechnology Information (NCBI) Short Read Archive (SRA) database (Accession Number: PRJNA1006674).

#### 3.6.1 Alpha- and beta diversity

**Figure 3:**
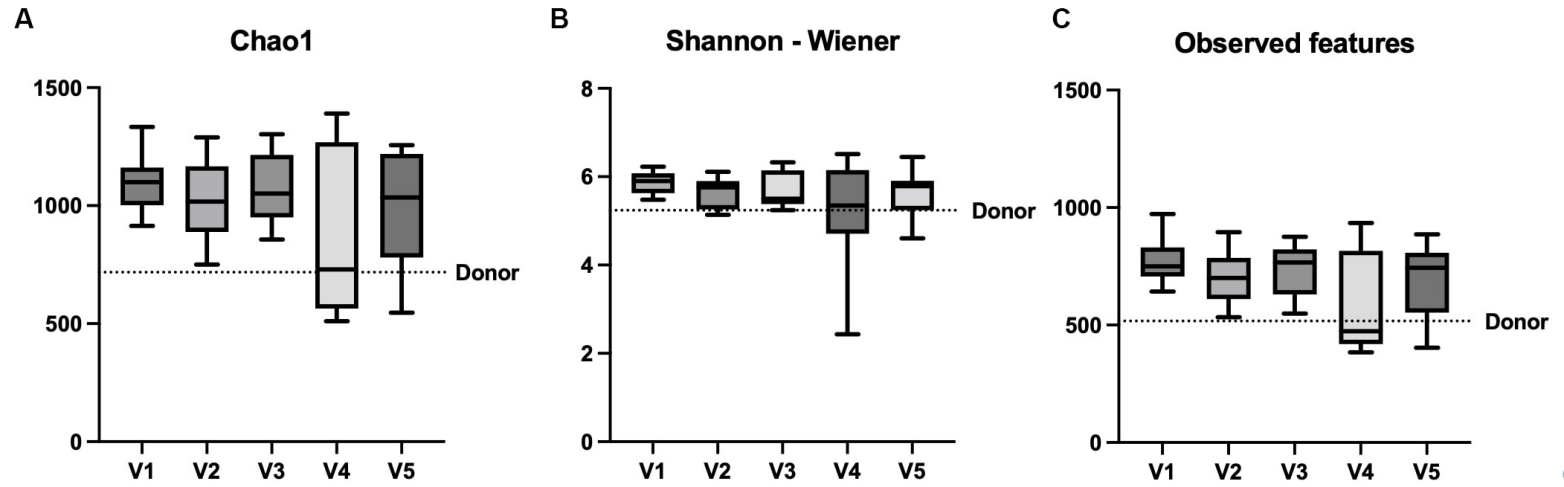
Comparison of alpha diversity parameters in box and whisker plots. The graphs show the comparison between V1, V2, V3, V4, and V5 **(A)** Chao1, **(B)** Shannon-Wiener index and **(C)** observed features. A transverse line in each box represents the median of each visit and the boxes represent the interquartile range, while the whiskers represent the minimum and maximum of the data. The dotted line represents the donor’s value. *p < 0.05 (Friedman test).

**Figure 4:**
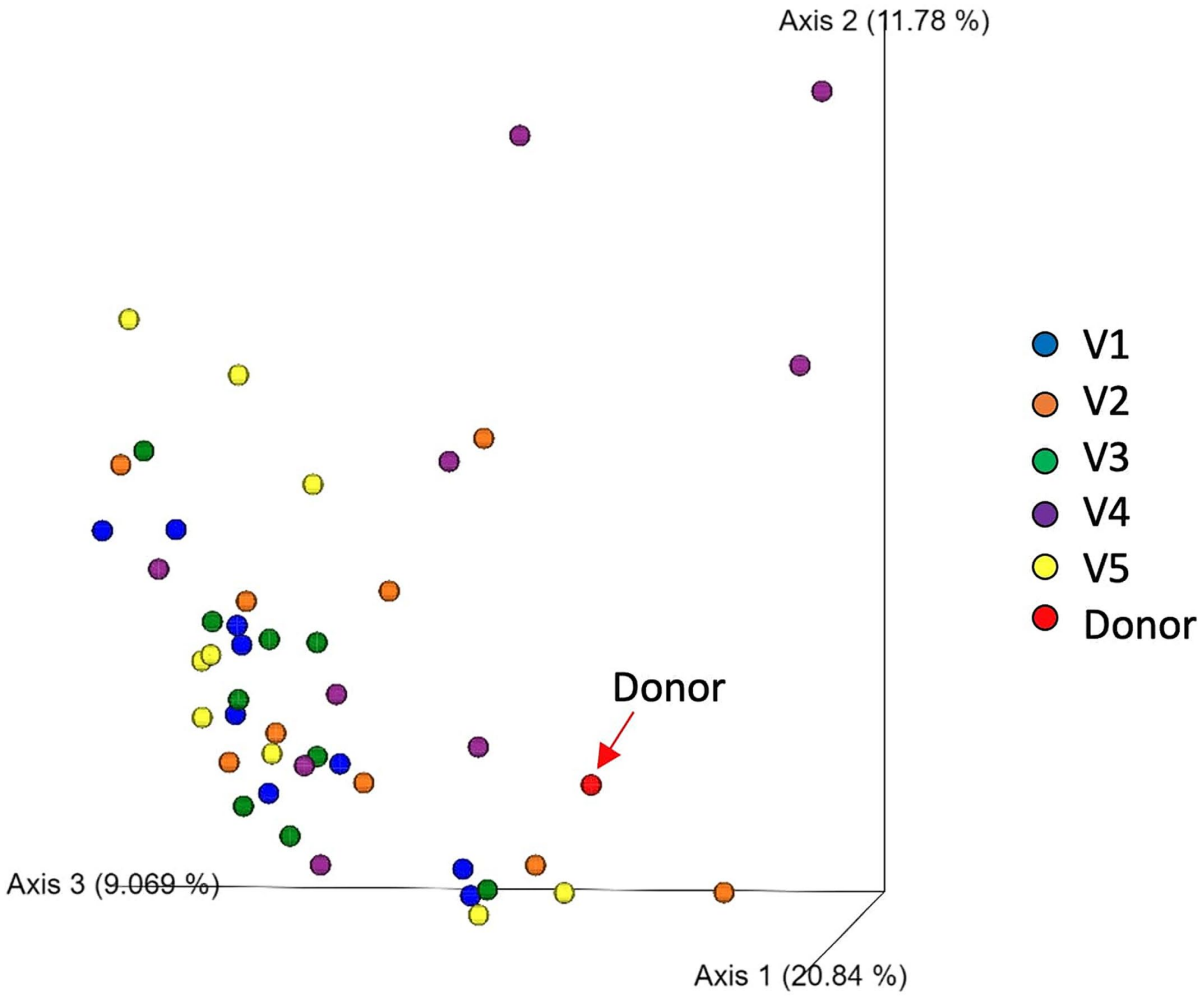
Principal coordinates analysis (PCoA) of weighted UniFrac distances of taxa diagrams demonstrating beta diversity of fecal samples of 9 dogs from V1 (blue), V2 (orange), V3 (green), V4 (purple), V5 (yellow) and the donor (red).

The alpha diversity including Chao1, Shannon-Wiener index and observed features remained unchanged after FMT (Figure 3). Beta diversity analysis showed no significant microbiome clustering between all dogs from each visit and the donor (ANOSIM; *R* = − 0.032, *p* = 0.809). In addition, there were no tendencies for microbiome clustering changes in the direction toward the donor after each FMT and the whole intervention (Figure 4).

#### 3.6.2 Taxonomic difference

**Figure 5:**
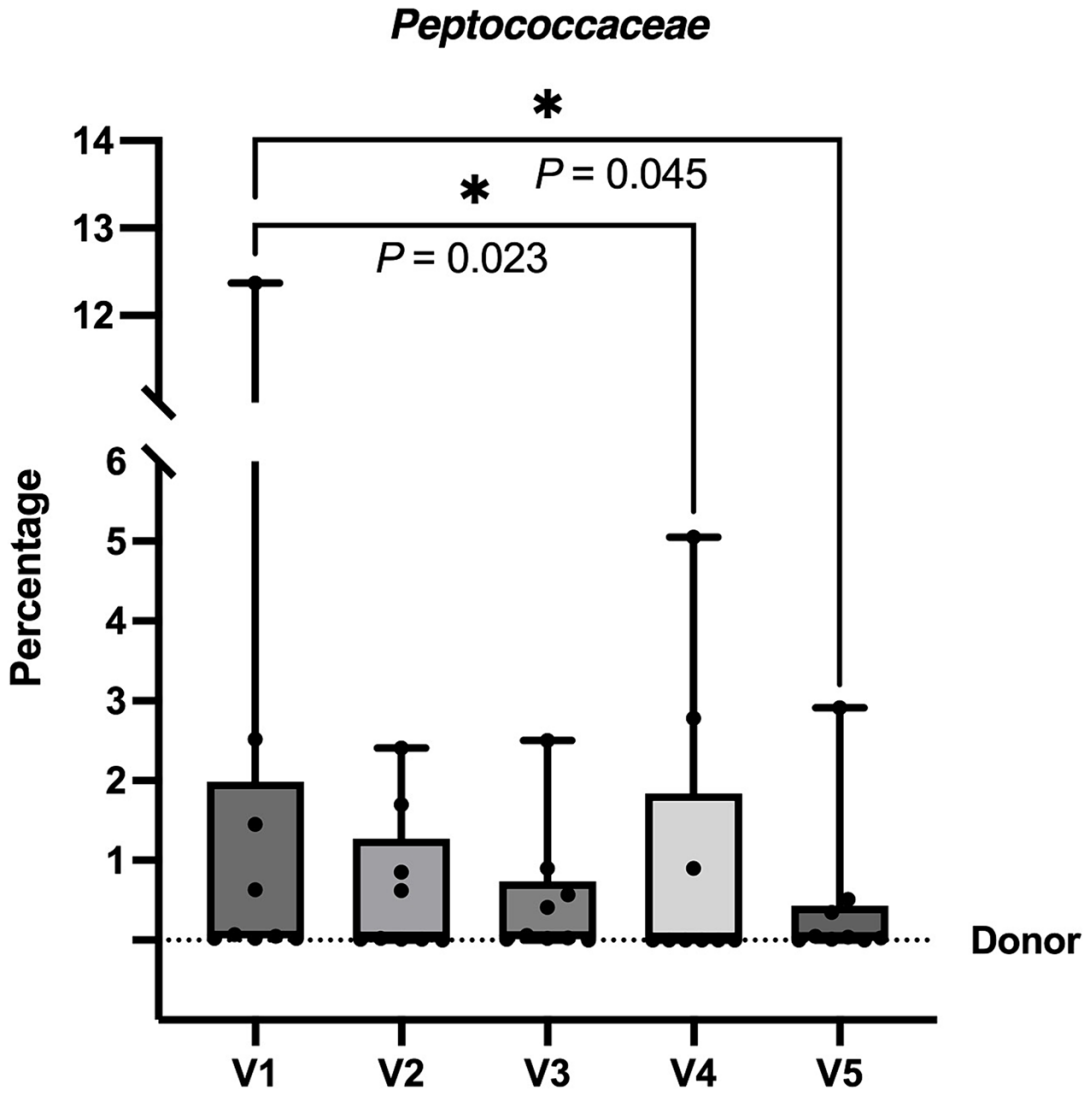
The graph illustrates the percentage of Family *Peptococcaceae* in the fecal samples of dogs before and after FMT from V1 to V5. The percentage levels of V4 and V5 were significantly lower than V1, indicating a reduction in the abundance of this family. Each box plot displays the median as a line and the interquartile range as the box. The whiskers represent the minimum and maximum values of the data. The dotted line represents the donor’s value. * *p* < 0.050 (Friedman test followed by Dunn test).

**Figure 6:**
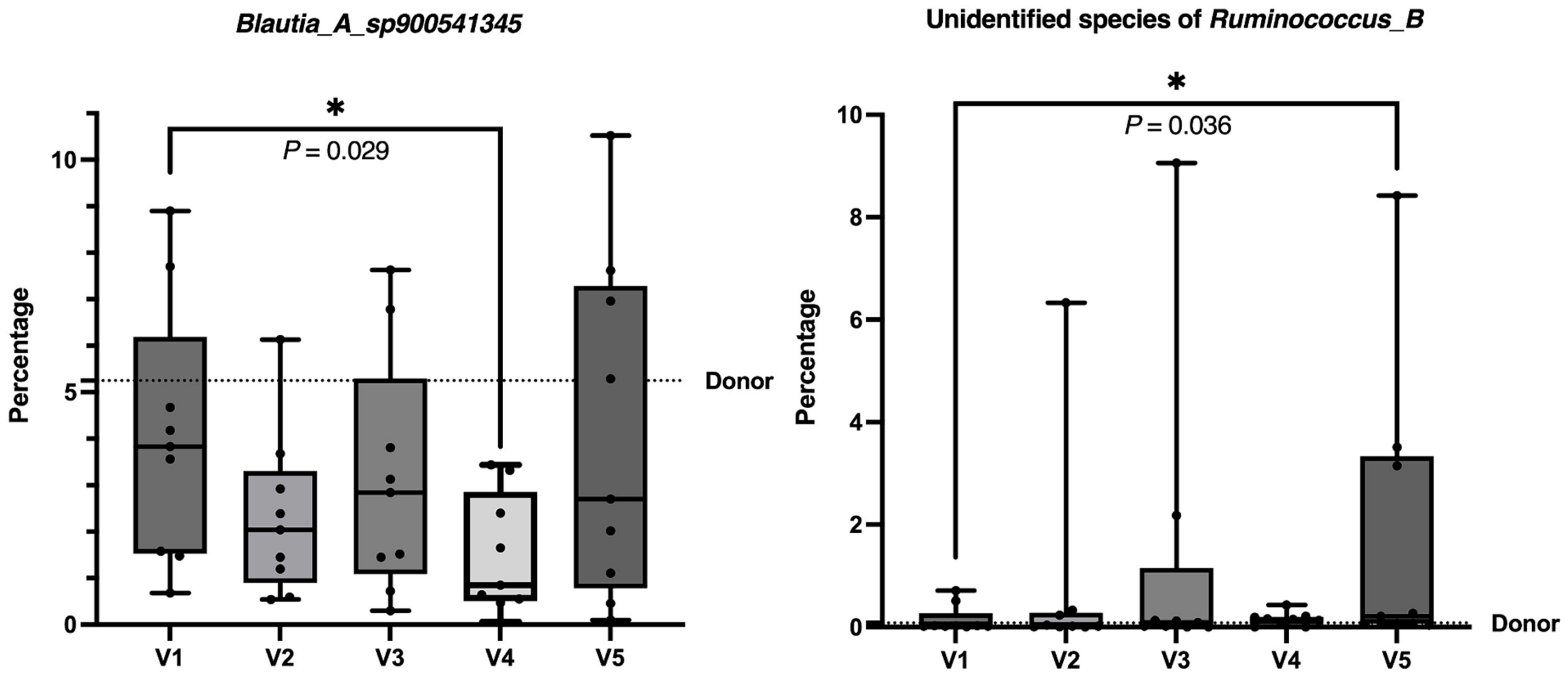
The graphs demonstrate the percentage of *Blautia_A_sp900541345* species and unidentified species of *Ruminococcus_B* in the fecal samples of dogs before and after FMT from V1 to V5. There was a decrease in Blautia_A_sp900541345 species at V4 and an increase in unidentified species of *Ruminococcus_B* at V5 compared to the baseline at V1. Each box plot represents the median as a line and the interquartile range as the box. The whiskers represent the minimum and maximum values of the data. The dotted line represents the donor’s value. *p < 0.050 (Friedman test followed by Dunn test).

In comparison to the baseline (V1), there were decreases in abundance of Firmicutes bacteria, namely in the phylum Firmicutes B, class Peptococcia, order Peptococcales, family *Peptococcaceae*, genus *UMGS1590* and species *UMGS1590 sp900553245* at V4 (*p* = 0.023) and V5 (*p* = 0.045) (Figure 5). Moreover, there was a decrease in *Blautia_A_sp900541345* species at V4 (*p* = 0.029) and an increase in unidentified species of *Ruminococcus_B* (*Ruminococcus_B_Snoopy_p1_metabat2_high_ PE.024.contigs*) at V5 (*p* = 0.036) (Figure 6).

#### 3.6.3 Functional gene analysis

The comparisons of the functional gene concentrations before and after FMT revealed no statistical changes.

#### 3.6.4 Dysbiosis index (DI)

In line with the results of metagenomic analysis, there were no significant changes comparing the DI from each visit to the V1 baseline. All samples were within reference intervals with DI < 0 at all time points (25).

#### 3.6.5 Short-chain fatty acid (SCFA) analysis

The comparison of SCFA concentrations in fecal samples from V1 to V5 demonstrated no significant changes of acetate, propionate, butyrate, isobutyrate, valerate, isovalerate, as well as total SCFA concentrations.

### 3.7 Urine neurotransmitters analysis

**Figure 7:**
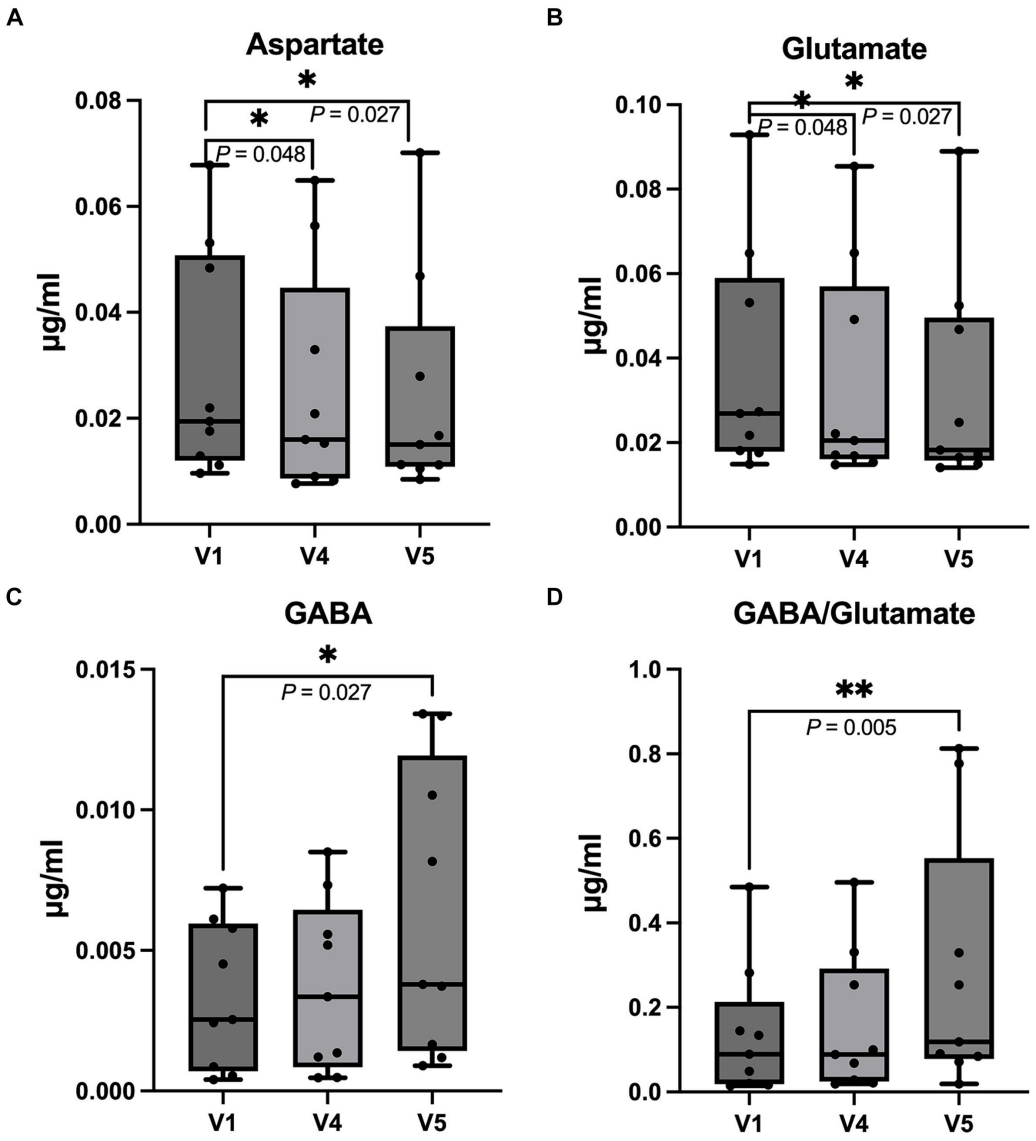
Box and whisker plots demonstrate a comparison of the urinal neurotransmitter concentration at the first appointment before FMT (V1) with the fourth (V4, three months after FMT) and fifth follow-up time point (V5, six months after FMT). (A) Aspartate; there was a decrease in aspartate concentration at V4 and V5 (B) Glutamate; the glutamate concentration was decreased at V4 and V5 compared to V1. (C) GABA; the GABA concentration at V5 was increased compared to V1. (D) the GABA/glutamate ratio; the graph shows an increase in the GABA/glutamate ratio at V5 compared to V1. The horizontal line in each box represents median, while the boxes represent the interquartile range. The whiskers represent the full range of data and the dots represent individual values (Wilcoxon signed-rank test, *p < 0.050, **p < 0.010).

The urine neurotransmitter concentration at V4 (three months after last FMT) and V5 (six months after last FMT) were compared to V1 (baseline). The aspartate concentration was significantly decreased at V4 (*p* = 0.048) and V5 (*p* = 0.027). The glutamate concentration was decreased at V4 (*p* = 0.048) and V5 (*p* = 0.027). In contrast, there were increases in the GABA concentration (*p* = 0.027) and the GABA/glutamate ratio (*p* = 0.005) at V5 (Figure 7). The concentration of noradrenaline, adrenaline, dopamine, serotonin, 5-hydroxytryptophan, glycine, serine, and glutamine remained unchanged throughout V1, V4, and V5.

### 3.8 Seizure frequency and severity

#### 3.8.1 Seizure frequency, seizure day frequency and seizure cluster days frequency

None of the dogs was seizure free after FMT. Only one of the nine dogs had a partial response (more than 50% reduction in seizure frequency) at 3 and 6 months follow up (dog no. 4 and 5 respectively). For cluster seizures, one (dog no. 4) and four dogs (dog no. 2,4,7, and 8) had more than 50% reduction in seizure cluster days frequency at 3 and 6 months follow-up, respectively (Supplementary file 5).

#### 3.8.2 Preictal-, ictal-, postictal and Interictal phases

No changes were reported during the preictal phase for any of the dogs. During the ictal phase, six out of nine dogs experienced *a* > 50% reduction in seizure duration, and in the post-ictal phase, a > 50% reduction in duration was observed in four out of nine dogs.

### 3.9 Side effects

Two dogs had mild, short-term side effects post-FMT. One displayed restlessness, and the other experienced abdominal bloating. These effects occurred after a single FMT and lasted for 1 day only.

## 4 Discussion

The link between the microbiome and behavior in humans and rodents has been shown in various studies, but data on how the microbiome can modify behavioral comorbidities in a naturally occurring canine model for epilepsy have not been studied to date. The current study suggested that FMT has the potential to ameliorate behavioral comorbidities, including ADHD- as well as fear- and anxiety-like behavior, in recipient dogs with DRE. Overall, owners reported an improvement in their dogs’ and their own quality of life.

Behavioral phenotype of the donor dog was successfully transferred by FMT to the dogs in the study group, leading to improvement in ADHD-like behavior. These findings align with preclinical rodent studies demonstrating that FMT could transfer behavioral phenotypes such as depression, anxiety-like behavior, and stress (12, 13). There is, however, limited data on the mechanistic pathways. In our study, the abundance of the phylum Firmicutes_B in the GIM, including the family *Peptococcaceae*, decreased after FMT. *Peptococcaceae* has been associated with stress and anxiety, as demonstrated in studies involving rats exposed to crowding stress and chicks experiencing heat stress (33). Similarly, higher maternal prenatal anxiety in humans has been linked to increased abundances of unidentified genera in the family *Peptococcaceae* (34). Therefore, the decrease in the abundance of *Peptococcaceae*, found in our study, may potentially have contributed to the alleviation of stress and anxiety.

We also found a decrease in *Blautia_A_sp900541345* and an increase in the unidentified bacteria in the genus *Ruminococcus_B* following FMT. In our study, we used GTDB taxonomy, which has different classifications compared to the taxonomy from NCBI. The GTDB aims to create a standardized microbial taxonomy based on genome phylogeny. The sequence-based phylogenetic trees offer a structure for crafting a taxonomy that considers both evolutionary connections and variations in evolutionary rates. Existing microbial taxonomies, such as those offered by NCBI, frequently diverge from evolutionary relationships due to the inclusion of polyphyletic groupings (35). The *Blautia_A_sp900541345* includes some bacteria from NCBI taxonomy, namely, *Blautia* sp. as well as bacteria from the family *Lachnospiraceae* and the order Clostridiales. Previous studies have reported an overgrowth of *Blautia* in ADHD patients (36), along with positive correlations between *Blautia* overgrowth and the severity of anxiety and depression in humans (37). In a preclinical study, rats with chronic unpredictable mild stress-induced anxiety-like and depression-like behavior also had a higher abundance of *Blautia* compared to the antibiotic-treated group, which showed a reduction in the behaviors (38). These studies showed that the abundance of *Blautia* had a positive correlation with the severity of psychiatric or behavioral problems in humans and animals, respectively. Therefore, the reduction of *Blautia* found in our study could be related to behavioral improvement. Nevertheless, some controversy exists. One study revealed that bacteria from the family *Lachnospiraceae* were decreased in ADHD patients (39) and this was associated with a reduction in repetitive behavior in a mouse model of autism spectrum disorder (40). No neuropsychological research data exists for Clostridiales and bacteria in the genus *Ruminococcus_B*. Regarding our outcomes, taxonomic changes in this study were minor, with unchanged functional genes, and dogs maintained normal DI. It is plausible that the link between the GIM and behavior encompasses intricate and finely differentiated factors, potentially including minute substances such as small metabolites or byproducts of the GIM, either with direct or indirect functions. It should also be taken into account that the reduced abundance of *Blautia* spp. found in our study did not last long, as it could only be detected at 3 months and not at 6 months after FMT. Moreover, the increase of *Ruminococcus_B* took time, as it could only be detected at 6 months after FMT. Observations over more than 6 months and a larger sample size could show how they change in the longer term and provide clearer results, respectively. It could be possible that a higher dose or more frequent FMT is needed to maintain steady abundance levels or provide more significant results in dogs with DRE.

The results of the questionnaires showed improvements in ADHD-, fear-, and anxiety-like behavior by reducing impulsivity, chasing, and non-social fear factors, as well as enhancing the quality of life of dogs and their owners. The outcomes were supported objectively by validated behavioral tests followed by computational analyses. The dogs in our study were calmer and showed less impulsivity after FMT. They walked less erratically and covered shorter distances than before FMT. In general, dogs with ADHD-, fear-, and anxiety-like behavior are expected to exhibit frequent rapid movements and turns, as well as high level of exploration behavior (23, 41). It should be taken into consideration that behavioral assessment requires comprehensive evaluation, particularly through clinical and objective analysis, to avoid bias from testers and owners. Further investigations are warranted into parameters that exhibit a strong correlation with specific behaviors.

Neurotransmitters have been found to be associated with psychiatric conditions. Emerging clinical research is starting to emphasize the significance of disrupted excitatory/inhibitory balance in ADHD and anxiety, associated with functional irregularities in glutamate and GABA signaling within the brain (42, 43). Several studies in humans using magnetic resonance spectrography reported an increase in glutamate or decrease in GABA concentrations in the specific brain areas such as anterior cingulate cortex of ADHD patients (44), thalamus of patients with social anxiety disorder (45), and occipital cortex of major depressive disorder patients (46). These studies highlighted the potential role of GABA and glutamate levels in neuropsychiatric disorder etiology. While specific brain area neurotransmitters may not directly correlate with total GABA and glutamate concentrations in cerebrospinal fluid, some disorders have shown similar directional changes. For example, there was an elevation of glutamate levels in cerebrospinal fluid of patients with depression (47) and obsessive compulsive disorder (48), whereas GABA was reported to decrease in patients with panic disorder (49) and anxiety, which also correlated with its severity (50). In our study, FMT could decrease the excitatory neurotransmitters aspartate and glutamate while increasing the inhibitory neurotransmitter GABA as well as GABA/glutamate ratio in urine. The improvement in the observed behaviors could be explained if the reduced glutamate and elevated GABA levels in urine reflect the corresponding levels in the brain or they could act peripherally via an unidentified mechanism such as vagal nerve activity modulation. This means, the neurotransmitter concentrations found in urine might not positively correlate with the concentrations of the neurotransmitters in the central nervous system, since neurotransmitters might not cross the blood–brain barrier and might not pass through peripheral systemic circulation before entering the kidneys and being present in urine (30). Moreover, the neurotransmitters could be influenced by neurotransmitter production from other organs, the GIM and nutrition (30). Therefore, the results should be interpreted with caution. Nevertheless, a correlation between peripheral and central neurotransmitters has been reported in some studies (51, 52) and changes in glutamate and GABA in plasma seemed to reflect their levels in the brain and cerebrospinal fluid (47, 53, 54). In addition, a study in epileptic dogs revealed a correlation of urinary neurotransmitter patterns and epilepsy (30). In this case, the improvement in ADHD and fear- and anxiety-like behaviors found in our study might be explained by the reduction of glutamate and increase in GABA levels. The exact mechanism by which these neurotransmitters contribute to these disorders remains uncertain, and they may not be fully explained by a single neurotransmitter system (55). Further studies on neurotransmitters using magnetic resonance spectroscopy in dogs with behavioral comorbidities should provide more information and may support our results.

Many drugs can affect the GIM. When selecting donors, it’s crucial to consider their medications, particularly antibiotics, gastroprotectants, and non-steroidal anti-inflammatory drugs. Antibiotics, for instance, can lead to long-term changes in the GIM, including reduced microbial diversity, depletion of beneficial bacteria, proliferation of harmful pathogens, and alterations in metabolic functions and byproducts (56). Other medications, like nonsteroidal anti-inflammatory drugs or gastroprotective drugs such as omeprazole, should also be taken into account (57, 58). In this study, a donor with well-controlled IE at Tier II confidence level, treated solely with PB, was chosen over a healthy donor without epilepsy due to the expected abundance of a transformed GIM. Generally, differences in the GIM between dogs with IE and healthy dogs have been observed (59). Furthermore, dogs with IE treated with PB exhibited differences in GIM metabolic functions, and products, such as higher production of SCFA, particularly in PB-responsive dogs (29). These differences may be related to varied responses of enteric neurons in dogs with IE (60). Therefore, the donor with well-controlled IE under PB treatment, providing the expected transformed GIM and associated products in fecal materials, was selected for use in this study. However, using healthy dogs as donors could yield different outcomes in terms of behavior and seizure control, potentially providing valuable information for donor selection.

This study has all the limitations of an open label study and can only be seen as a pilot study. Placebo effects are notable and should not be underestimated. They greatly influence outcomes in various medical conditions, as demonstrated by a meta-analysis on canine epilepsy, showing a 29% decrease in seizure frequency during placebo administration (61). Therefore, caution is needed when interpreting positive results in behavior, quality of life questionnaires, and seizure control data. However, these findings were reinforced by standardized behavioral tests and computational analysis. The example also exists in the study, improvements in seizure severity and frequency reported in the EpiQoL questionnaire are not reflected in statistical analysis. Another limitation is that the DNA analyses via shotgun sequencing underwent repeated freeze–thaw cycles, which could potentially impact minor changes in the GIM (62). The study’s small sample size underscores its pilot nature. Future investigations with larger sample sizes and control groups are warranted for more robust evidence. The decision to conduct a pilot study was influenced by the absence of prior indications of FMT’s impact on canine behavior associated with epilepsy and offers the advantage of estimating effect size.

## 5 Conclusion

This pilot study provides the first evidence that FMT could be considered as one of the procedures to improve behavior in dogs with DRE. In this study, FMT showed a clear effect on behavior and urinary neurotransmitters. Overall, owners reported an improved quality of life for themselves and their dogs with epilepsy. Additionally, seizure severity and frequency of tonic–clonic seizures only improved in individual dogs. Randomized controlled studies are urgently needed to confirm the initial promising results, which could also have important translational value for comorbidities in human epilepsy.

## Data availability statement

The datasets presented in this study can be found in online repositories. The names of the repository/repositories and accession number(s) can be found below: https://www.ncbi.nlm.nih.gov/, PRJNA1006674.

## Ethic statement

The animal studies were approved by the Lower Saxony State Office for Consumer Protection and Food Safety, Germany (LAVES). The studies were conducted in accordance with the local legislation and institutional requirements. Written informed consent was obtained from the owners for the participation of their animals in this study.

## Author contributions

AW: Writing – review & editing, Writing – original draft, Project administration, Methodology, Investigation, Formal analysis, Data curation, Conceptualization. SM: Writing – review & editing, Validation, Supervision, Methodology, Investigation, Conceptualization. NF: Writing – review & editing, Software, Methodology, Investigation, Formal analysis. JS: Writing – review & editing, Validation, Supervision, Methodology, Formal analysis, Conceptualization. RP: Writing – review & editing, Formal analysis, Data curation. MK: Writing – review & editing, Formal analysis. BL: Writing – review & editing, Formal analysis, Data curation. AB-N: Writing – review & editing, Resources. AF: Writing – review & editing, Supervision, Resources, Methodology, Conceptualization. KB-H: Writing – review & editing, Methodology. CF: Writing – review & editing, Resources, Methodology. MG: Writing – review & editing, Methodology, Formal analysis. FR: Writing – review & editing, Validation, Supervision, Methodology, Formal analysis. AZ: Writing – review & editing, Software, Methodology, Formal analysis. HV: Writing – review & editing, Validation, Supervision, Methodology, Conceptualization.

## Footnotes

^1^www.tech4animals.org
