# Supplementary material for: Behavioral comorbidities treatment by fecal microbiota transplantation in canine epilepsy: a pilot study of a novel therapeutic approach
Source: Front Vet Sci. 2024 Jun 21;11:1385469. doi: 10.3389/fvets.2024.1385469 (PMC11229054; doi:10.3389/fvets.2024.1385469)
Supplement: Supplementary file 1 [file Data_Sheet_1.pdf]

Supplementary file 1: seizure seminology questionnaire

1. How old was your dog, when he/she had the first seizure?

▢ < 1 year old ▢ 2 years old ▢ 3 years old ▢ 4 years old
▢ 5 years old ▢ 6 years old

2. When did your dog have the last seizure?

▢ In the last 24 hours
▢ In the last 7 days
▢ In the last 30 days
▢ 1-3 months ago
▢ > 3 months ago

3. What does the seizure of your dog look like?

▢ Generalized seizure of the whole body without consciousness and with or without urination or defecation
▢ Focal seizure such as twitching of one of the body parts such as face, lip, eye, or leg.
▢ Starting with a focal seizure before developing to a generalized seizure

4. Which signs did your dog have before the seizure (preictal phase)?

▢ Hiding/ isolation
▢ Nervous
▢ Staying close/ needs attention
▢ Restlessness
▢ Fear
▢ Whining
▢ Shivering
▢ Hypersalivation
▢ Tiredness/ being sleepy
▢ Sniffing
▢ Hungry
▢ Thirsty
▢ Completely normal - none of the signs mentioned above

5. Which signs did your dog have after the seizure (postictal phase)?

▢ Disorientation
▢ Ataxia
▢ Staring blankly in the air
▢ Clingy/ needs attention
▢ Complete or partial vision loss
▢ Tiredness/ being sleepy
▢ Fear
▢ Aggression
▢ Restlessness
▢ Sniffing

▢ Hungry
▢ Thirsty
▢ Completely normal - none of the signs mentioned above

6. How many seizures did your dog have in the last three months?
▢ 1
▢ 2-5
▢ 6-10
▢ 10-20
▢ > 20

7. How long did the average seizure last?
▢ < 1 min
▢ 1-2 min
▢ 3-5 min
▢ 6-10 min
▢ > 10 min

8. Has your dog ever had a seizure that lasted more than 5 min (status epilepticus)?
▢ Yes
▢ No, always less than 5 min

9. Has your dog ever had at least two seizures in 24 hours (cluster seizure)?
▢ Yes
▢ No, only one seizure in 24 hours

10. If yes, how many seizures did your dog have in 24 hours?
▢ 2 seizures
▢ 3-5 seizures
▢ > 5 seizures
