# Supplementary material for: Behavioral comorbidities treatment by fecal microbiota transplantation in canine epilepsy: a pilot study of a novel therapeutic approach
Source: Front Vet Sci. 2024 Jun 21;11:1385469. doi: 10.3389/fvets.2024.1385469 (PMC11229054; doi:10.3389/fvets.2024.1385469)
Supplement: Supplementary file 2 [file Data_Sheet_2.pdf]

<u>Supplementary 2: questionnaire for FMT effects and side effects</u>

1. What is the name of your dog?

2. What is your name?

3. Did your dog experience seizures during the last two weeks?
Please choose one of the following answers:
☐ No, my dog didn´t have any seizures during the last two weeks.
☐ Yes, my dog had seizures during the last two weeks.

4. If "yes", how many seizures did your dog experience during the last two weeks?
Please choose one of the following answers:
☐ 1
☐ 2
☐ 3
☐ 4
☐ >5

5. Are your dog's seizures different at the moment regarding, for example, length, intensity and character?
Please choose one of the following answers:
☐ No, there is no difference
☐ Yes (please describe) ____

6. Did your dog have the following symptoms after receiving fecal transplantations?
Please choose the most suitable answers:
☐ Normal, none of the symptoms stated below
☐ Abdominal discomfort/pain
☐ Bloating
☐ Flatulence
☐ Constipation
☐ Diarrhea
☐ Vomiting
☐ Low fever
☐ High fever
☐ Restlessness/pacing
☐ Itching
☐ Reddened skin
☐ Other: (please describe) ____

7. When did these symptoms start?
Please choose one of the following answers:
☐ Immediately after fecal transplantation
☐ 2-3 days after fecal transplantation
☐ 4-5 days after fecal transplantation
☐ One week after fecal transplantation
☐ Two weeks after fecal transplantation

8. How long did the symptoms last?
Please choose one of the following answers:
- ▢ The dog had the symptoms for only one day
- ▢ The symptoms lasted a few days
- ▢ The symptoms lasted roughly one week
- ▢ The symptoms lasted approximately two weeks

9. After which fecal transplantation did your dog have these symptoms?
Please choose the most suitable answers:
- ▢ After the first fecal transplantation
- ▢ After the second fecal transplantation
- ▢ After the third fecal transplantation

10. Did the symptoms become stronger or weaker after every consecutive fecal transplantation?
Please choose one of the following answers:
- ▢ Symptoms became stronger
- ▢ Symptoms remained the same
- ▢ Symptoms became weaker
- ▢ Intensity of the symptoms varied often

11. Does or did your dog show uncommon behavior after fecal transplantations?
Please choose one of the following answers:
- ▢ No, my dog behaves as he did before.
- ▢ Yes, the behavior of my dog is a little better than before.
- ▢ Yes, the behavior of my dog is a little worse than before.
- ▢ Yes, the behavior of my dog completely changed and is much better than before.
- ▢ Yes, the behavior of my dog completely changed and is much worse than before.

If "yes", please describe how the behavior of your dog worsened:

____

12. How would you score the feces of your dog?
Please choose one of the following answers:
▢ 1 ▢ 1.5 ▢ 2 ▢ 2.5 ▢ 3. ▢ 3.5 ▢ 4 ▢ 4.5 ▢ 5

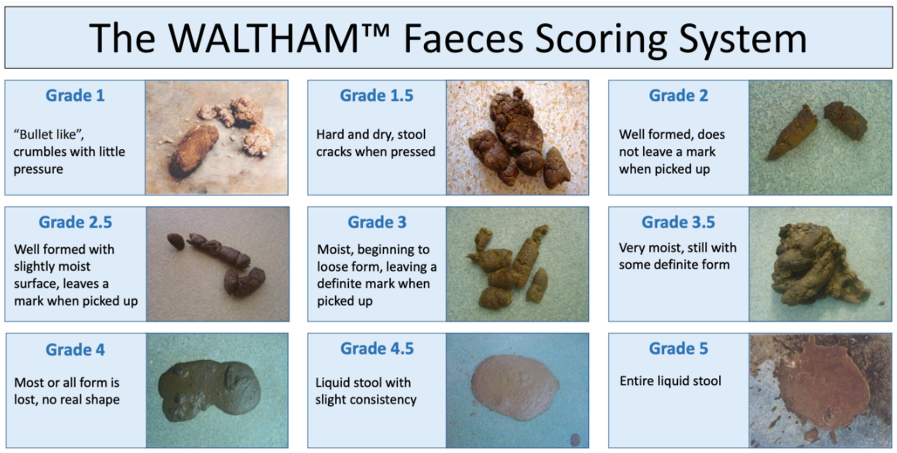

From Waltham™
