# Supplementary material for: Behavioral comorbidities treatment by fecal microbiota transplantation in canine epilepsy: a pilot study of a novel therapeutic approach
Source: Front Vet Sci. 2024 Jun 21;11:1385469. doi: 10.3389/fvets.2024.1385469 (PMC11229054; doi:10.3389/fvets.2024.1385469)
Supplement: Supplementary file 3 [file Data_Sheet_3.pdf]

Supplementary file 3: behavioral tests

The behavioral tests included anxiety and cognition tests. The tests were performed in a 4x4 m testing room with some furniture and recorded by five cameras.

1. Anxiety test

The anxiety test included three tasks: open field (new environment), separation- and stranger-directed fear test, and open field (thunderstorm).

Task 1 Open field (new environment):

Exploratory behavior in an unfamiliar place was assessed. The owner had the dog enter the room (Figure 1A). The tester sat on chair 1, while the owner stood in the center of the room and unleashed the dog (starting time point) before sitting on chair 2. The tester talked with the owner for 1 min. During this period, the dog was allowed to explore the room without any restriction. After finishing this task, chair 1 was moved to the center of the room and chair 2 was removed (Figure 1B). The owner sat on chair 1.

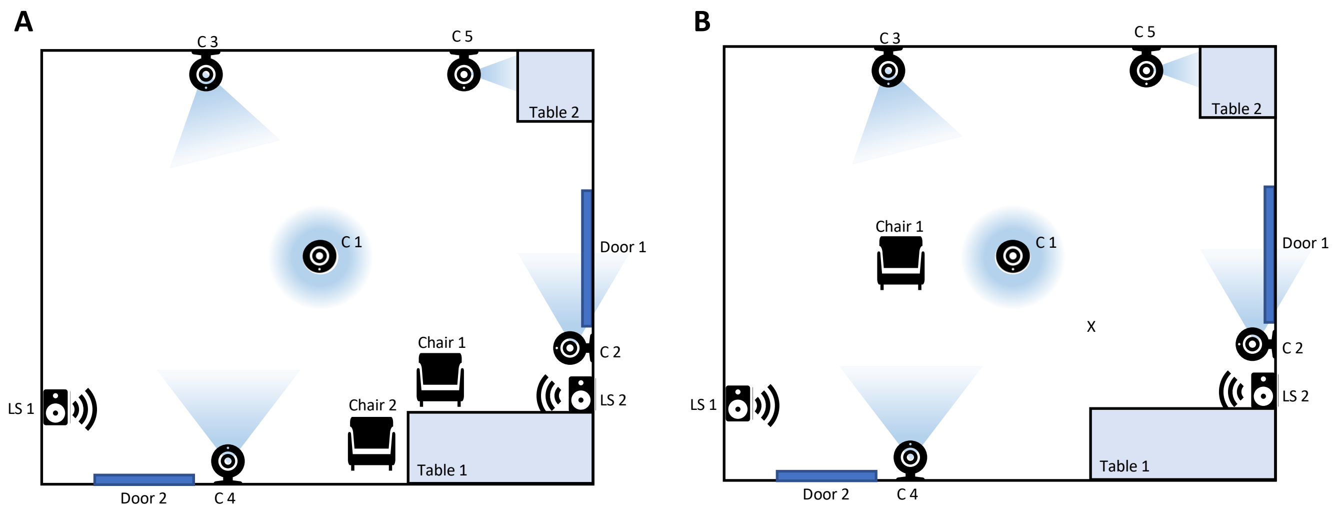

Figure 1A-B: The figure demonstrates the top view of the behavioral testing room (A) during open field (new environment) test and (B) separation- and stranger-directed-fear test, as well as open field (audio) test. In this room, five cameras (C1-C5) were mounted on the ceiling and walls. The C1 was attached to the middle of the ceiling to get an overhead view of the room and linked to a tablet outside the room for monitoring. C2, C3, and C4 were attached to the walls to record the side views. Table 2 was covered with a table cloth, which allowed the dog to hide under the table (table 1 was a block side table). The space under table 2 was filmed using C5, which was attached to one side of a wall. C5 evaluated the dog's reaction, in case the dog was scared and hid under the table. Two loudspeakers (LS1, LS2) mounted on the ceiling were used for the noise challenge in the audio test. The cross on the floor shown in B is the position of the stranger in phases 2 and 3 of the anxiety test (see Section 2.7.1 and Supplementary file 3 for more details).

Task 2: Separation- and stranger-directed fear test

The aim of this test was to evaluate the dog's possible separation anxiety and the reaction to a stranger. This test consisted of five phases.

- Phase 1: Owner and dog – The owner stayed in the room with the dog for 1 min. During this period, the owner sat on chair 1 and was allowed to look at the dog and talk to it but not allowed to touch it.
- Phase 2: Owner, stranger, and dog – The stranger entered the room through door 1 and stood between the owner and the door for 1 min without interacting with the dog.
- Phase 3: Stranger and dog – The owner was instructed to leave the room through door 1 by the tester who gently knocked on the door from outside. This phase was started when the owner closed the door. The dog was left with the stranger for 1 minute. The stranger could play and/or interact with the dog if the dog was willing to do so. If the dog was distressed, the stranger could make attempts to provide comfort.
- Phase 4: Dog alone – The stranger left the room. This phase started when the stranger closed the door. The dog was left alone for 2 minutes. At the end of this phase, the owner went behind door 2 and called the dog's name twice with 5 seconds apart to distract the dog inside the room from door 1 before walking to and entering door 1.
- Phase 5: Owner and dog (reunion) – The owner entered the room through door 1 and greeted the dog intensively for 15 sec. At the end of this phase, the owner sat on chair 1.

Task 3 Open field (thunderstorm)

While the owner sat on the chair, the recording of a thunderstorm was played for 3 min in order to assess the sound-induced fear and anxiety behavior of the dog. The sound measured at the center and around the room was controlled and kept at 90 decibels.

Video-based automatic behavior analysis

The three anxiety test tasks were analyzed using K9-Blyzer (Canine Behavior Analyzer), a tool for automatic video analysis of canine behaviors, which has already been used for a variety of scientific projects [1-4]. Blyzer's architecture is comprised of two layers: (a) A computer vision layer that uses a neural network model for object detection, with the object being dog, or dog and person depending on the task, and (b) an analysis, or sense-making module that identifies and quantifies the requested parameters from the spatio-temporal data (trajectory) obtained from module (a). The architecture of Blyzer is depicted in Figure 2.

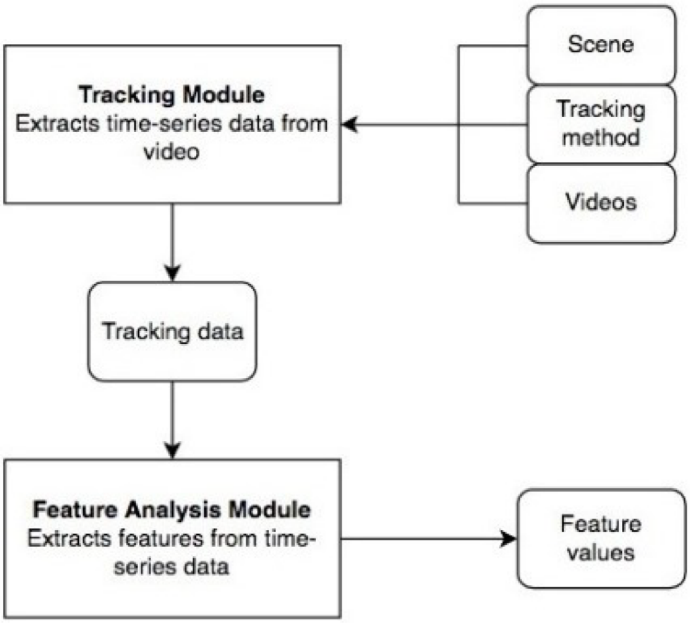

Figure 2. Blyzer's architecture

In the configuration of this paper, the tracking method input was a neural network based on the Faster R-CNN architecture [5]. The neural network was retrained on images of dogs and people to improve detection. The video input consisted of videos featuring either an open field (new environment) task, an open field (thunderstorm) task, or a phase from the separation and stranger-directed fear test. The input scene included one static (non-moving) object, door 1, and 1-3 dynamic objects (the participating dog, the owner, the stranger). Depending on the test, dynamic objects are detected by the tracking method. For each video, the system provided (a) the participating dog's trajectory and (b) the calculated parameters described in Table 1. Examples of object detection and trajectories are shown in Figure 3.

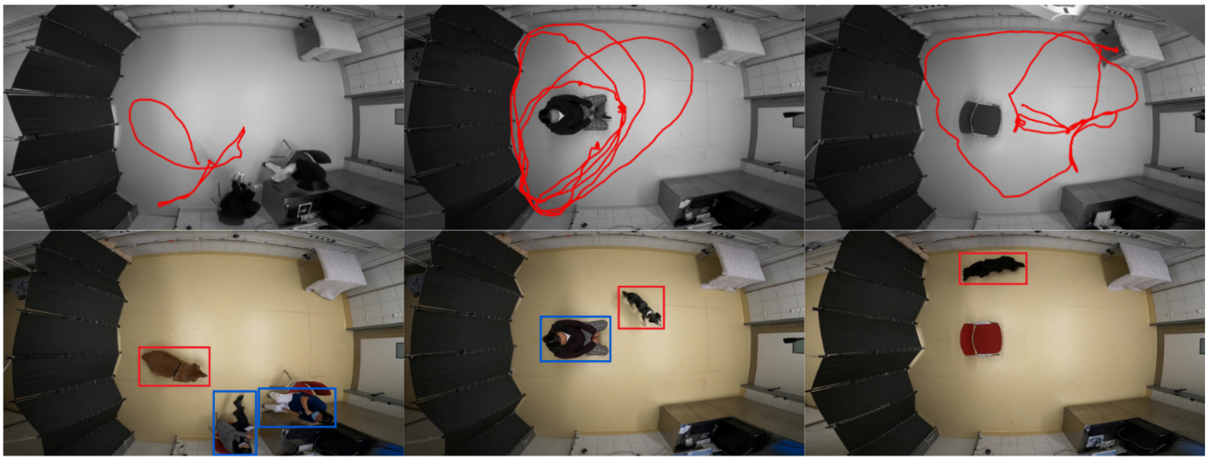

Figure 3: Top row: participating dog's trajectory extracted with Blyzer; bottom row: frames with the participating dog, owner, and stranger being tracked. Left: open field (new environment); middle and right: separation and stranger-directed fear test phases 1 and 4, respectively.

Quality of detection. To ensure sufficient tracking, only videos with a percentage of frames where dog and person (where appropriate) were correctly detected in at least 80% of the frames were used, leading to the exclusion of one video. For the remaining videos, post-processing. operations available in BLYZER were applied to remove noise and enhance detection quality using smoothing and extrapolation techniques for the detected objects, reaching almost perfect (above 98%) detection.

Table 1: Parameters used in the analysis of anxiety tests using K9-Blyzer software.

Variable	Explanation	Units
TD	Total distance of movement of the dog	cm
Turn0_30	Number of turns between 0 and 30 degrees	times
Turn30_60	Number of turns between 30 and 60 degrees	times
Turn60_90	Number of turns between 60 and 90 degrees	times
Turn90_120	Number of turns between 90 and 120 degrees	times
Turn120	Number of turns greater than 120 degrees	times
Area	Polygon area of the dog's convex hull movement	cm^2
IU	Intensity of use or the ratio between total movement and the square root of the area of movement	percentage
ST	Straightness net displacement distance divided by the total length of dog's movement	
Pace	Ratio between time and total distance the dog moved	s/cm
Avg. speed	Dog's average speed	cm/s
% OCST_O	Percentage of time spent outside a circle around the owner of 1m radius	percentage
Avg. Dis_O	Average distance from owner	cm
% OCST_S	Percentage of time spent outside a circle around the stranger of 1m radius	percentage
% duration near door 1	Time dog spent in a 1m radius circle around door 1	percentage

2. Cognition test

The cognition test consisted of two tasks including a spatial working memory task and a problem-solving task. The tasks were performed in the same behavioral lab as the anxiety test, but the room was totally cleared. The tasks were based on the modified protocol from a study by Winter and others [6], which was modified from the original published validated protocol of González-Martínez and others [7] in order to assess spatial working memory and problem-solving ability. The exact times were also quantified and compared. The scoring system of both tasks is described in Table 2.

Task 1: Spatial working memory test

The owner had the dog on a leash at the center of the room (Figure 4). The tester stood 60 cm away from the dog in front of it and showed the dog the piece of sausage for 2 seconds. The tester maintained eye contact with the dog, while he walked to one of the room corners and placed the sausage on the floor. The tester left the room through door 1. Then, the owner left the room with the dog through door 2 and stayed outside for 15 seconds before reentering the room and standing in the center of the room. The owner unleashed the dog and let it search for the treat without any commands. The test started when the dog was unleashed and ended when the dog found the food or the food was not found within one minute. The test was repeated three times. Each time, the food was repositioned in the new corner in the same order for every dog.

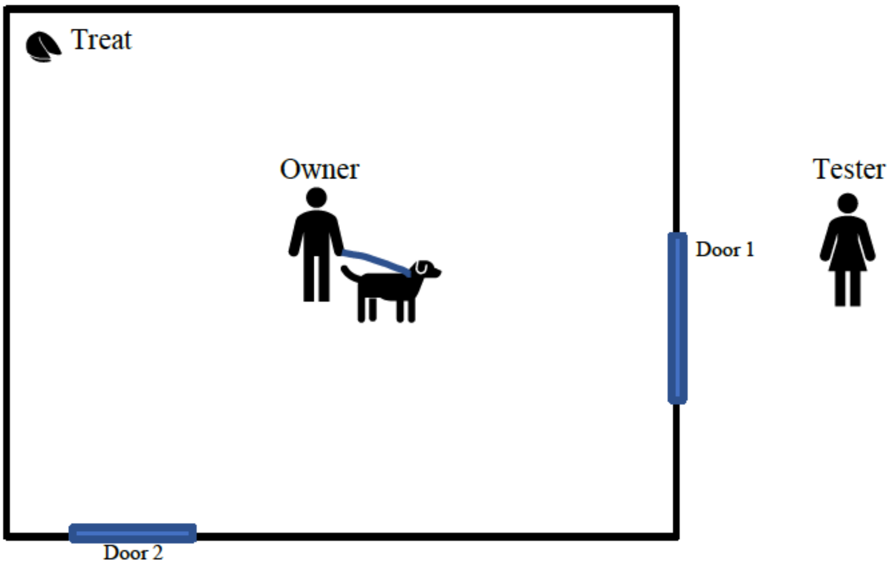

Figure 4: The figure demonstrates the top view of the behavioral test room of the cognition test.

Task 2: Problem-solving test

A piece of sausage was placed under a transparent box at the center of the room. The size of the box depended on the dog's size; body weight < 10 kg: small box (55 g), 10-20 kg: medium box (96 g), > 20 kg: large box (160 g). The time was recorded from unleashing the dog until either the dog managed to get the food or did not manage to get the food within 2 min. The test was repeated three times.

Table 2: The scoring system modified from Winter and others [6] used in task 1 and 2 of the cognition test.

Score	Task 1 (spatial working memory)	Task 2 (problem solving ability)
1	Goes directly towards the food.	Successfully gets the food within 2 minutes.
2	Finds the food within one minute.	Did not manage to get the food within 2 minutes.
3	Searches for the food for one minute without success.	Sniffs the box but does not try to get the food.
4	Makes no attempt to search for the food.	Makes no attempt to get the food.

References

1. Zamansky, A.; Sinitca, A.; Linden, D.v.d.; Kaplun, D. Automatic Animal Behavior Analysis: Opportunities for Combining Knowledge Representation with Machine Learning. *Procedia Computer Science* **2021**, *186*, 661-668, doi:https://doi.org/10.1016/j.procs.2021.04.187.
2. Fux, A.; Zamansky, A.; Bleuer-Elsner, S.; van der Linden, D.; Sinitca, A.; Romanov, S.; Kaplun, D. Objective Video-Based Assessment of ADHD-Like Canine Behavior Using Machine Learning. *Animals (Basel)* **2021**, *11*, doi:10.3390/ani11102806.
3. Bleuer-Elsner, S.; Zamansky, A.; Fux, A.; Kaplun, D.; Romanov, S.; Sinitca, A.; Masson, S.; van der Linden, D. Computational Analysis of Movement Patterns of Dogs with ADHD-Like Behavior. *Animals (Basel)* **2019**, *9*, doi:10.3390/ani9121140.
4. Menaker, T.; Monteny, J.; de Beeck, L.O.; Zamansky, A. Clustering for Automated Exploratory Pattern Discovery in Animal Behavioral Data. *Front Vet Sci* **2022**, *9*, 884437, doi:10.3389/fvets.2022.884437.
5. Ren, S.; He, K.; Girshick, R.; Sun, J. Faster R-CNN: Towards real-time object detection with region proposal networks. *IEEE transactions on pattern analysis and machine intelligence* **2016**, *39*, 1137-1149.
6. Winter, J.; Packer, R.M.A.; Volk, H.A. Preliminary assessment of cognitive impairments in canine idiopathic epilepsy. *Vet Rec* **2018**, *182*, 633, doi:10.1136/vr.104603.
7. Gonzalez-Martinez, A.; Rosado, B.; Pesini, P.; Garcia-Belenguer, S.; Palacio, J.; Villegas, A.; Suarez, M.L.; Santamarina, G.; Sarasa, M. Effect of age and severity of cognitive dysfunction on two simple tasks in pet dogs. *Vet J* **2013**, *198*, 176-181, doi:10.1016/j.tvjl.2013.07.004.
