# Supplementary material for: Behavioral comorbidities treatment by fecal microbiota transplantation in canine epilepsy: a pilot study of a novel therapeutic approach
Source: Front Vet Sci. 2024 Jun 21;11:1385469. doi: 10.3389/fvets.2024.1385469 (PMC11229054; doi:10.3389/fvets.2024.1385469)
Supplement: Supplementary file 4 [file Data_Sheet_4.pdf]

Supplementary file 4: analysis of urine neurotransmitters

The urine samples were thawed at room temperature for 15 min before analysis.

1. Monoamine neurotransmitters

The neurotransmitter standards used were dopamine (H8502, Sigma-Aldrich), noradrenaline (A0937, Sigma-Aldrich), adrenaline (E4375; Sigma-Aldrich), serotonin (H7752, Sigma-Aldrich), and 5-Hydroxy-L-tryptophan (H9772, Sigma-Aldrich), which is a serotonin precursor.

Twenty µL of each urine sample was analyzed via HPLC (Shimadzu, Kyoto, Japan) with an octadecylsilyl (ODS) column (Nucleosil 5 C18 250 x 4.6 mm; Macherey-Nagel GmbH & Co. KG, Düren, Germany) using fluorescence detection (Ex 285 nm, Em 330 nm) at 35 °C. The mobile phase consisted of 100 mL methanol and 900 mL of 0.15 mM sodium acetate buffer at pH 4. The process lasted for 50 min.

2. Amino acid neurotransmitters

The neurotransmitter standards used were glutamate (G1251, Sigma-Aldrich), GABA (A2129, Sigma-Aldrich), aspartate (A9256, Sigma-Aldrich), glycine (G7126, Sigma-Aldrich), serine (S4500, Sigma-Aldrich), and glutamine (G3126, Sigma-Aldrich), which is a glutamate precursor.

OPA solution was freshly prepared on the day of the analysis by dissolving 1 mL OPA (Sigma-Aldrich) in 2 µL Mercapthoethanol (LiChrosolv®, Merck KGaA, Darmstadt, Germany). For the urine sample preparation, 200 µL of each urine sample was mixed with 1800 µL ultra-pure water and filtered through a 0.45 µm sterile cellulose acetate membrane filter (VWR International, LLC, Radnor, PA, USA). After filtration, 50 µL of the filtered mixed samples were diluted with 450 µL ultra-pure water. Then, 50 µL of each prepared sample was mixed with the 50 µL OPA solution, followed by 10 µL of 6 % phosphoric acid before being injected into the HPLC using an ODS column with fluorescence detection (Ex 330 nm Em 445 nm) at 35 °C. The mobile phase consisted of 270 mL methanol, 5 mL tetrahydrofuran, and 0.1 M sodium acetate buffer. The process took 50 min.
