# Supplementary material for: Behavioral comorbidities treatment by fecal microbiota transplantation in canine epilepsy: a pilot study of a novel therapeutic approach
Source: Front Vet Sci. 2024 Jun 21;11:1385469. doi: 10.3389/fvets.2024.1385469 (PMC11229054; doi:10.3389/fvets.2024.1385469)
Supplement: Supplementary file 5 [file Data_Sheet_5.pdf]

Supplementary 5: The table shows information about each patient including signalment, nutrition, medications and their dosages, serum phenobarbital (PB), and potassium bromide (KBr) concentration (conc.) before FMT (V1) and at third and fifth visits (V3 and V5), supplements, history of status epilepticus (SE) and cluster seizures (CS) as well as tonic-clonic seizure frequency (SF), seizure days frequency (SDF) and seizure cluster days frequency (SCF) per month during the six-month retrospective period before fecal microbiota transplantation (FMT) and at the three month- (V4) and six-month follow-ups (V5) after FMT.

**Dog no.**	**Breed**	**Gender**	**Age**	**Age of IE onset**	**Nutrition**		**ASDs**	**Dosage**	**PB and KBr conc. (µg/ml)**			**Supple-ments**	**SE/CS history**	**Before FMT (times per month)**			**After FMT (times per month)**					
					**Diets**	**Treats**			**V1**	**V3**	**V5**			**6 months**			**3 months follow-up**			**6 months follow-up**		
														**SF**	**SDF**	**SCF**	**SF**	**SDF**	**SCF**	**SF**	**SDF**	**SCF**
1	Crossbreed (AS x CKCS)	Female neutered	8 yr 2 mths	6 mths	CDF from pork and poultry	beef and lamb treats	PB	8 mg/kg bid	37.1	35.1	33.8	-	-	2.00	2.00	0.00	3.67	3.67	0	2.33	2.33	0
							KBr	12 mg/kg bid	1340	1100	860											
							Lev	40 mg/kg tid	-	-	-											
2	Crossbreed (GR Mix)	Female neutered	3 yr 1 mth	1 yr 3 mths	CDF from lamb	beef treats, V	PB	3.8 mg/kg bid	37.5	29.1	36.1	-	CS	2.50	2.00	0.33	2.67	2.00	0.33	2.00	2.00	0
							KBr	11.4 mg/kg bid	1830	2110	1970											
3	Jack Russell	Male neutered	5 yr 6 mths	3 yrs 2 mths	CCF+CDF from poultry	beef and pork treats	PB	3.1 mg/kg bid	20.8	20.9	-	-	CS	7.50	1.83	1.67	Exclusion due to death					
							KBr	15 mg/kg tid	1060	976	-											
4	French Bulldog	Female intact	1 yr 5 mths	8 mths	CDF from poultry	-	PB	2.9 mg/kg bid	22.7	24.5	26.5	-	CS	4.83	2.50	1.50	2.33	2.00	0.33	3.00	2.33	0.67
							KBr	12.3 mg/kg bid	1352	1910	1480											
							Lev	15 mg/kg bid	-	-	-											
5	French Bulldog	Male intact	1 yr 2 mths	7 mths	CDF from duck and salmon	duck and deer treats	PB	4.7 mg/kg bid	26.6	27.8	34.6	-	CS	4.67	1.50	0.83	5.67	1.33	1.33	2.33	1.67	0.67
							KBr	35.8 mg/kg am<br>11.9 mg/kg pm	1150	1780	1310											
							Lev	30.3 mg/kg tid	-	-	-											
6	English Bulldog	Female intact	3 yrs 8 mths	2 yrs 2 mths	CCF from beef, V	horse treats	PB	6.8 mg/kg bid	24.3	32.6	33.3	CBD oil	CS	5.83	1.50	1.00	7.33	1.67	1.67	7.67	1.67	1.67
							Im	11.5mg/kg bid	-	-	-											
7	Crossbreed (AS x BMD)	Male neutered	5 yrs	3 yrs 1 mth	CDF from poultry, V	-	PB	5 mg/kg bid	32.4	32.0	30.3	MCT oil	CS	2.33	1.83	0.50	4.67	2.33	0.67	1.67	1.67	0
							KBr	14.8 mg/kg bid	1443	1820	1470											
							Im	10 mg/kg sid	-	-	-											
8	Crossbreed (AS x GS)	Male neutered	7 yrs 3 mths	3 yrs 2 mths	CCF+CDF from kangaroo	kangaroo treats	PB	2.7 mg/kg bid	30.8	21.5	18.4	MCT oil	SE, CS	3.00	1.17	0.83	2.67	0.67	0.67	2.67	2.67	0
							KBr	16 mg/kg sid am<br>16 mg/kg sid pm	1480	1180	1580											
							Lev	23 mg/kg qid	-	-	-											
9	Beagle	Female neutered	5 yrs 1 mth	4 yrs 1 mth	CDF from chicken	V	PB	3.6 mg/kg bid	32.4	33.9	25.6	-	CS	2.00	2.00	0.00	3.33	3.33	0	3.33	3.33	0
							KBr	17.7 mg/kg sid am<br>23.7 mg/kg sid pm	1120	1160	1490											
10	AS	Male neutered	9 yrs 4 mths	3 yrs	CDF from horse	V, F, M	PB	3.8 mg/kg bid	23.9	21.4	25.6	MCT and fish oil	CS	4.50	1.67	1.50	2.33	1.00	1.00	3.67	2.00	1.33
							KBr	12.4 mg/kg bid	1420	1420	1250											
							Lev	21.4 mg/kg tid	-	-	-											

IE; idiopathic epilepsy, ASDs; anti-seizure drugs, AS; Australian Shepherd, CKCS; Cavalier King Charles Spaniel, GR; Golden Retriever, BMD; Bernese Mountain Dog, GS; German Shepherd, yr; year-old, mth; month-old, CCF; commercial can food, CDF; commercial dry food, V; vegetables, F; fruits, M; milk products, Lev; levetiracetam, Im; imepitoin, sid; once a day; bid, 2 times a day, tid; 3 times a day, qid; 4 times a day, am; morning, pm; evening, CBD; cannabidiol, MCT; medium-chain triglycerides.
